# Clonal heterogeneity of *FLT3*-ITD detected by high-throughput amplicon sequencing correlates with adverse prognosis in acute myeloid leukemia

**DOI:** 10.18632/oncotarget.25729

**Published:** 2018-07-10

**Authors:** Katrin Schranz, Max Hubmann, Egor Harin, Sebastian Vosberg, Tobias Herold, Klaus H. Metzeler, Maja Rothenberg-Thurley, Hanna Janke, Kathrin Bräundl, Bianka Ksienzyk, Aarif M.N. Batcha, Sebastian Schaaf, Stephanie Schneider, Stefan K. Bohlander, Dennis Görlich, Wolfgang E. Berdel, Bernhard J. Wörmann, Jan Braess, Stefan Krebs, Wolfgang Hiddemann, Ulrich Mansmann, Karsten Spiekermann, Philipp A. Greif

**Affiliations:** ^1^ Experimental Leukemia and Lymphoma Research, Department of Medicine III, University Hospital, LMU Munich, Munich, Germany; ^2^ Laboratory for Leukemia Diagnostics, Department of Medicine III, University Hospital, LMU Munich, Munich, Germany; ^3^ German Cancer Consortium, partner site Munich, Germany; ^4^ German Cancer Research Center, Heidelberg, Germany; ^5^ Department of Medical Data Processing, Biometrie and Epidemiology, LMU Munich, Munich, Germany; ^6^ Institute of Human Genetics, University Hospital, LMU Munich, Munich, Germany; ^7^ Leukaemia and Blood Cancer Research Unit, Department of Molecular Medicine and Pathology, Faculty of Medical and Health Sciences, University of Auckland, Auckland, New Zealand; ^8^ Institute of Biostatistics and Clinical Research, University of Münster, Münster, Germany; ^9^ Department of Medicine A, Hematology and Oncology, University of Münster, Münster, Germany; ^10^ Division of Hematology and Oncology, Charité Universitätsmedizin Berlin, Campus Virchow, Berlin, Germany; ^11^ Department of Hematology and Oncology, Barmherzige Brüder Hospital, Regensburg, Germany; ^12^ Laboratory for Functional Genome Analysis, Gene Center, LMU Munich, Munich, Germany

**Keywords:** acute myeloid leukemia (AML), fms-related tyrosine kinase 3 (FLT3), iternal tandem duplication (ITD), next generation sequencing (NGS), fragment analysis

## Abstract

In acute myeloid leukemia (AML), internal tandem duplications (ITDs) of *FLT3* are frequent mutations associated with unfavorable prognosis. At diagnosis, the *FLT3-*ITD status is routinely assessed by fragment analysis, providing information about the length but not the position and sequence of the ITD. To overcome this limitation, we performed cDNA-based high-throughput amplicon sequencing (HTAS) in 250 *FLT3-*ITD positive AML patients, treated on German AML Cooperative Group (AMLCG) trials. *FLT3-*ITD status determined by routine diagnostics was confirmed by HTAS in 242 out of 250 patients (97%). The total number of ITDs detected by HTAS was higher than in routine diagnostics (*n* = 312 vs. *n* = 274). In particular, HTAS detected a higher number of ITDs per patient compared to fragment analysis, indicating higher sensitivity for subclonal ITDs. Patients with more than one ITD according to HTAS had a significantly shorter overall and relapse free survival. There was a close correlation between *FLT3-*ITD mRNA levels in fragment analysis and variant allele frequency in HTAS. However, the abundance of long ITDs (≥75nt) was underestimated by HTAS, as the size of the ITD affected the mappability of the corresponding sequence reads. In summary, this study demonstrates that HTAS is a feasible approach for *FLT3-*ITD detection in AML patients, delivering length, position, sequence and mutational burden of this alteration in a single assay with high sensitivity. Our findings provide insights into the clonal architecture of *FLT3-*ITD positive AML and have clinical implications.

## INTRODUCTION

Muations in the fms-related tyrosine kinase 3 (*FLT3*) gene are prevalent in newly diagnosed acute myeloid leukemia (AML) cases, affecting up to 39% patients. Internal tandem duplications (ITDs) represent the most common type of *FLT3* mutation, being most freuquent in patients with normal karyotype (cytogenetically normal AML, CN-AML) and in patients positive for the translocation t(6;9)(p23;q34) or t(15;17)(q22;q21) [1–4]. *FLT3*-ITD is associated with an unfavorable prognosis due to reduced duration of complete remsission (CR), shorter event free survival (EFS) and shorter overall survival (OS) [1, 5–7]. The European Leukemia Net included the *FLT3*-ITD mutation as prognostic risk factor into a clinical risk-stratification that may guide physicians in therapy decisions [8, 9]. Patients carrying *FLT3*-ITD mutations with high allelic ratio benefit from a more intensive consolidation treatment such as allogeneic stem cell transplantation [7, 10–12]. Since the recent approval of the tyrosine kinase inhibitor (TKI) Midostaurin in combination with induction chemotherapy, *FLT3*-mutated patients may profit from this targeted treatment. [13–15] *FLT3*-ITDs are predominately located in exon 14 and 15, affecting the juxtamembrane domain (JM) and tyrosine kinase domain 1 (TKD1) of the FLT3 receptor [16]. Depending on the *FLT3*-ITD insertion site and respective functional domain, differential outcome and response to treatment with conventional chemotherapy as well as TKIs were observed; especially non-JM ITDs displayed a resistance to TKIs [10, 16–21]. Although always leading to an in-frame transcript, *FLT3*-ITDs vary in length (between three to over 400 nucleotides (nt)) and sequence [1, 2, 4, 6, 17, 22, 23]. Whether the ITD length has an impact on outcome remains controversial [1, 22, 24, 25]. However, a prognostic relevance was observed for the mutant to wild-type (WT) allelic ratio which corresponds to the size of the clone(s) with the *FLT3*-ITD. A high allelic burden is associated with poor prognosis (shorter OS, EFS), and outcome is even worse for patients with a loss of WT *FLT3* [1, 4, 6, 16, 17, 26–29]. Additionally, up to five distinct clones with different *FLT3*-ITDs were observed per patient [16]. In light of these findings, the assessment of *FLT3*-ITD characteristics is of important prognostic value. In routine diagnostics, the *FLT3*-ITD status is assessed by capillary electrophoresis of PCR-amplified cDNA (hereafter referred to as ‘fragment analysis’) [26]. However, this assay only provides the length but not the position and the sequence of the insertion. Therefore, it is attractive to overcome these methodological limitations by the use of high-throughput amplicon sequencing (HTAS) as an alternative strategy for *FLT3*-ITD detection. Techniques based on next generation sequencing (NGS) have the potential to assess multiple parameters simutaneously with scalable sensitivity [27, 30–32]. Previous studies already highlighted the potential of *FLT3*-ITD detection by NGS, especially with regards to diagnosis and disease monitoring, e.g. minimal residual disease (MRD) detection, as evaluation of treatment response and early detection of relapse [16, 17, 27, 30–34]. Establishment of NGS assays in diagnostic routine requires high sensitivity at low costs as well as fast turn around time and reliable results. To evaluate the applicability and accuracy of NGS-based *FLT3*-ITD detection for routine diagnostics, we compared *FLT3*-ITD detection by HTAS and fragment analysis in 250 adult *FLT3*-ITD positive AML patients.

## RESULTS

### HTAS reliably identifies *FLT3*-ITD subclones of prognostic relevance

**Figure 1:**
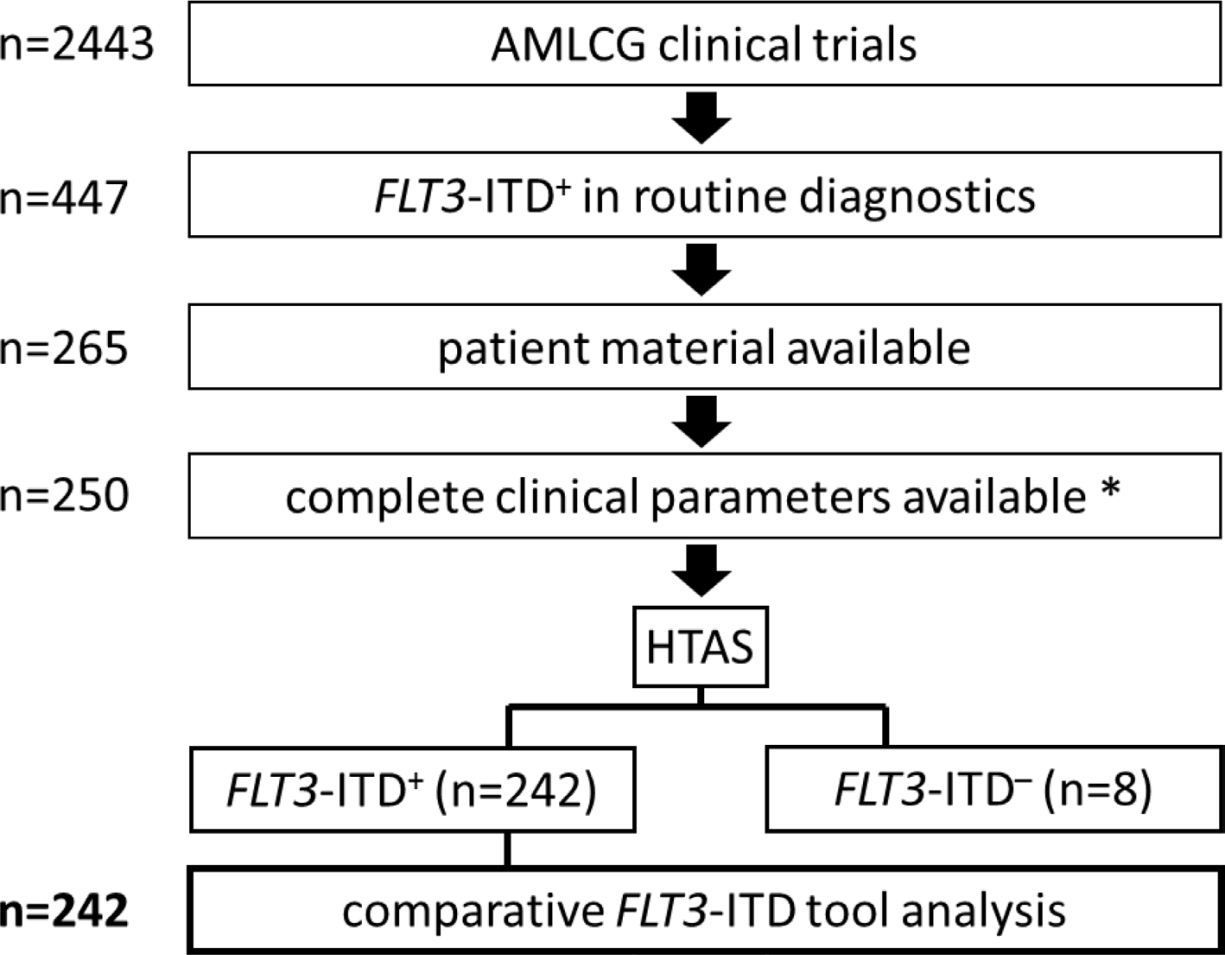
Study design Flow chart illustrating the selection of patient samples. ^*^Residual patients were lost during follow-up. HTAS (high-throughput amplicon sequencing), ITD (internal tandem duplication).

**Figure 2:**
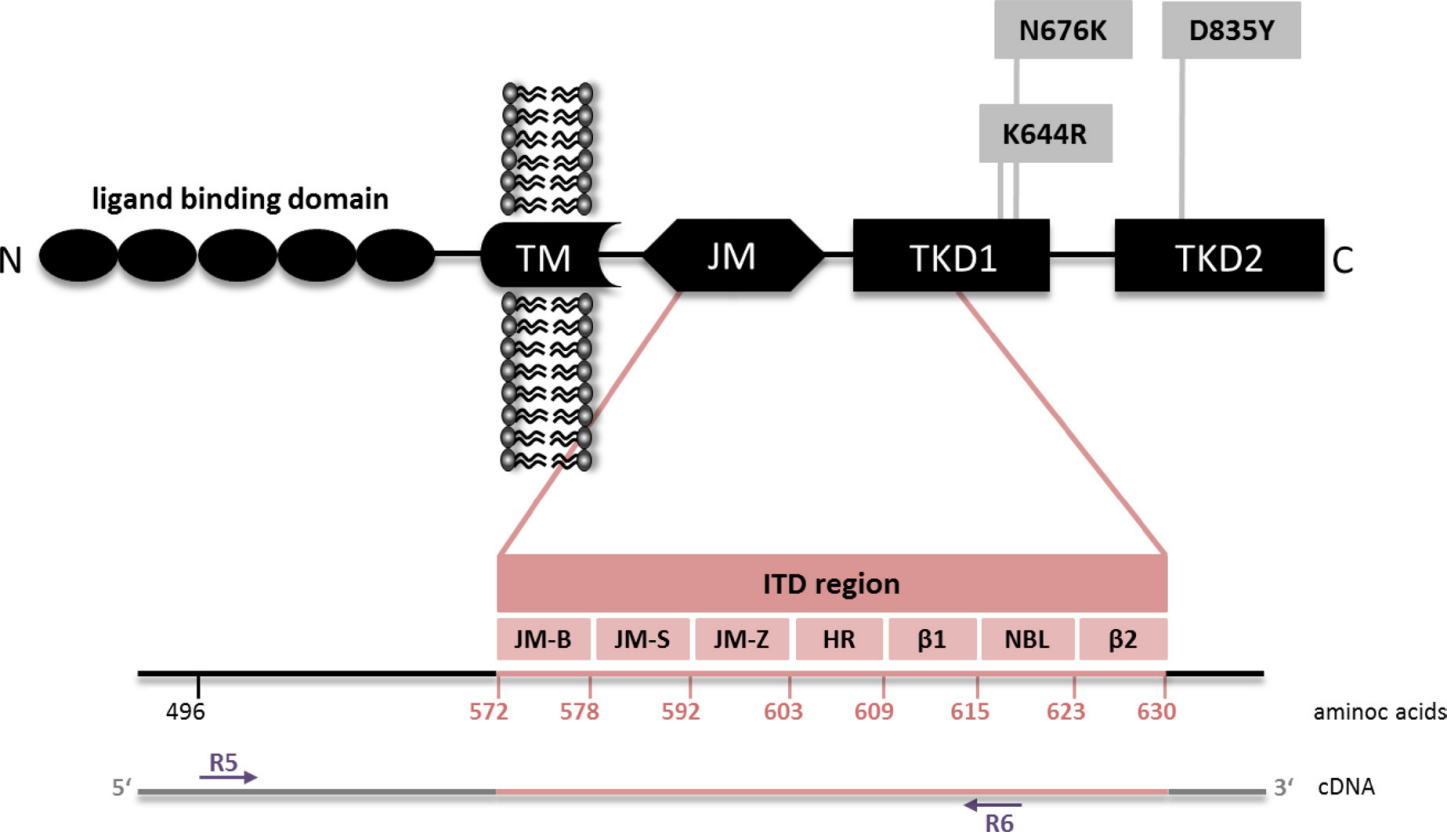
FLT3 mutations Schematic illustration displaying AML-specific FLT3 mutations according to their receptor domain localization (modified from Opatz *et al.* [72]). FLT3-ITDs are located within the juxtamembrane (JM) and tyrosine kinase domain (TKD) 1, whereas point mutations are frequently found in TKD1 and TKD2. *FLT3* cDNA region covered by HTAS and fragment analysis is indicated by the primer binding marks (R5 and R6). ITD (internal tandem duplication), JM-B (JM binding motif), JM-S (JM switch motif), JM-Z (JM zipper motif), HR (hinge region), β1 (β1-sheet), NBL (nucleotide binding loop), β2 (β2-sheet).

**Figure 3:**
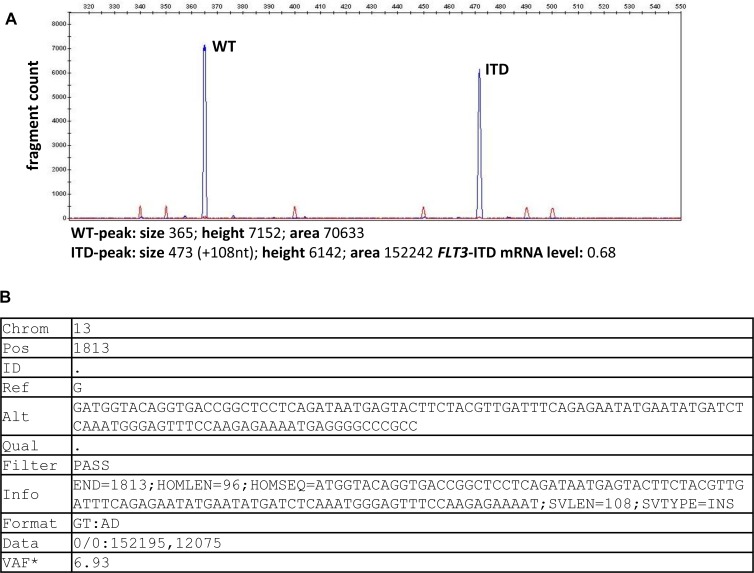
*FLT3*-ITD detection output by fragment analysis and by HTAS Exemplary results are shown for UPN 42 (**A)** Electropherogram displays *FLT3* amplicon signals as fragment peaks, with the distance of the peak positions corresponding to the size of the ITD and the area under the peak curves used for calculation of the mutational burden. (**B**) Tabular representation of variant detection results from HTAS in VCF format (Pindel output). WT (wild type), ITD (internal tandem duplication), Chrom (Chromosome), Pos (cDNA position), Ref (reference sequence), Alt (alternative sequence), VAF (variant allele frequency) ^*^computed separately and added manually.

**Figure 4:**
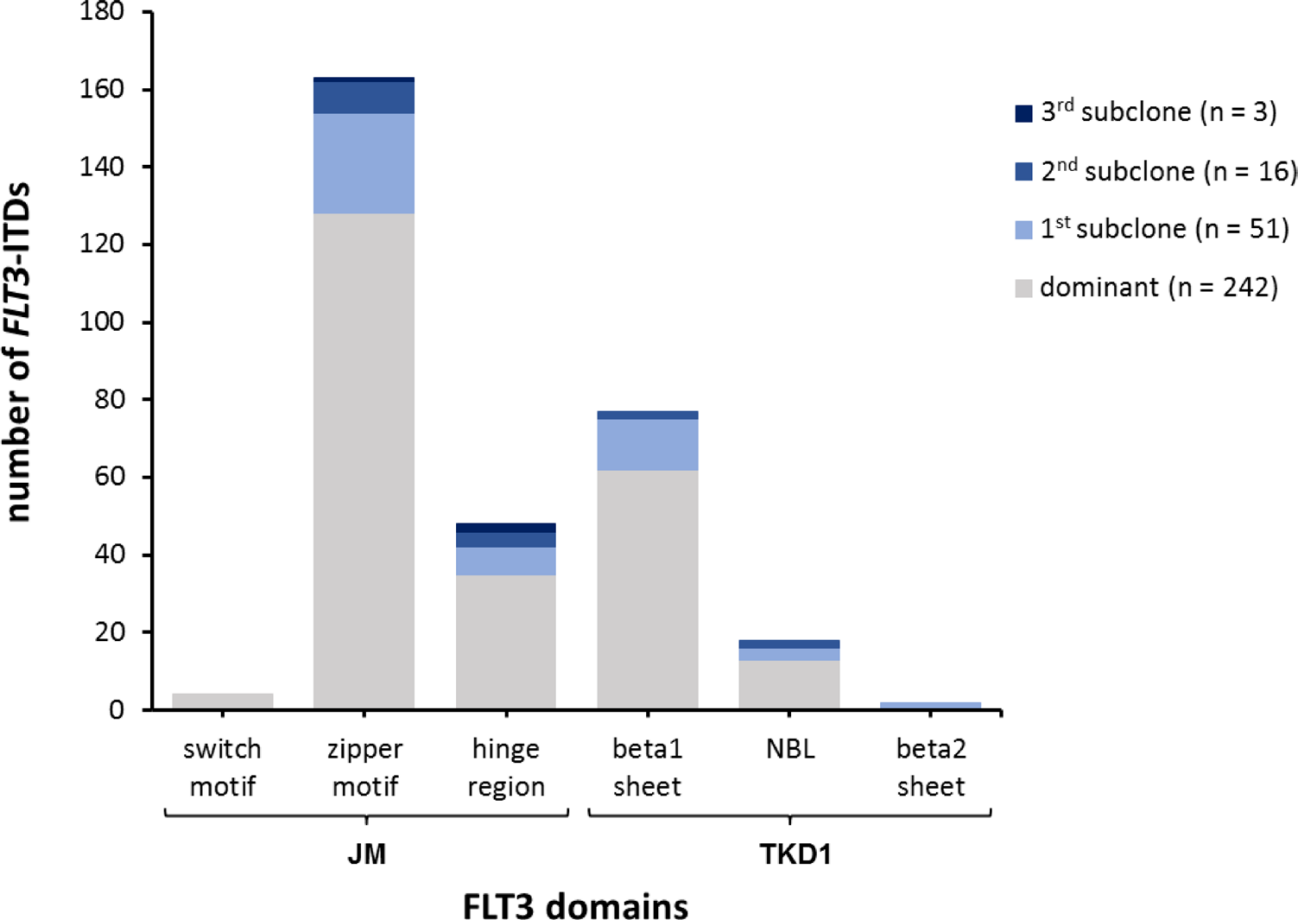
FLT3-ITDs assigned to functional domains Distribution of detected *FLT3*-ITDs by HTAS across functional domains according to insertion site and clone size. ITD (internal tandem duplication), JM (juxtamembrane), TKD (tyrosine kinase domain), NBL (nuclear binding loop).

**Figure 5:**
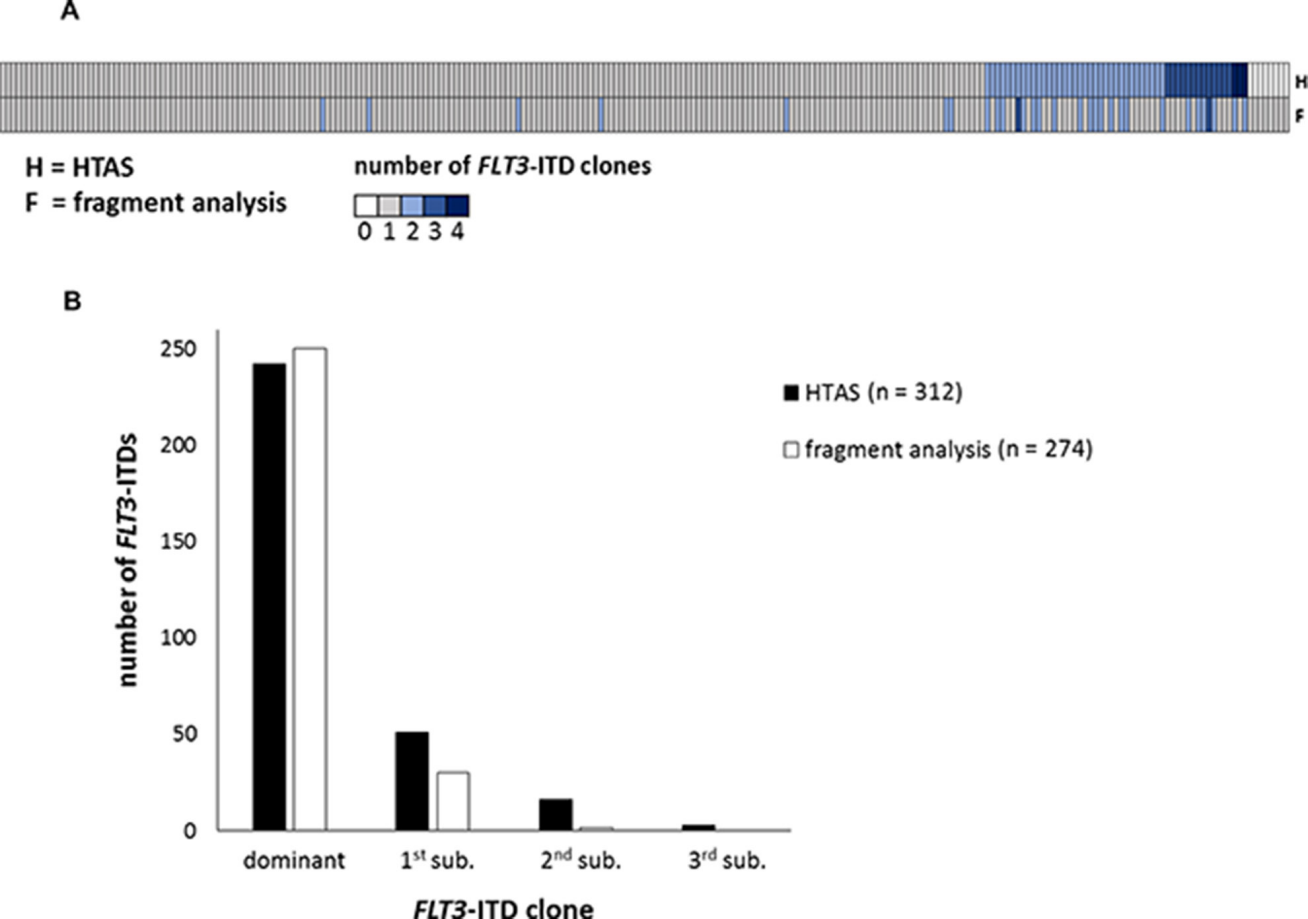
*FLT3*-ITD subclone detection (**A**) Number of detected *FLT3*-ITDs per patient (*n =* 250), comparing HTAS to fragment analysis. (**B**) Number of detected *FLT3*-ITDs per method, comparing HTAS to fragment analysis according to clonal size. HTAS (high-throughput amplicon sequencing), ITD (internal tandem duplication), sub (subclone).

**Figure 6:**
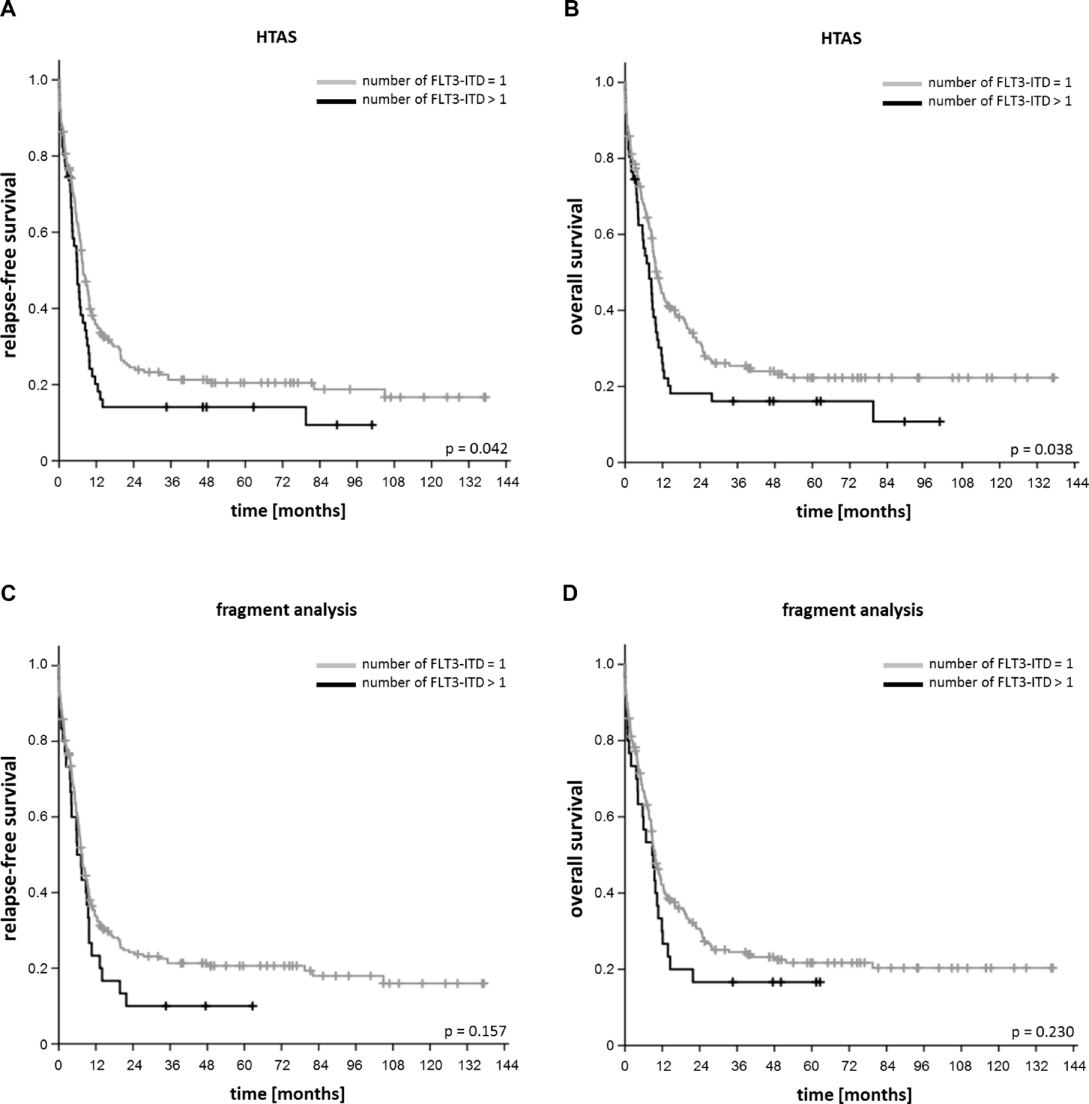
Impact of the number of *FLT3*-ITD clones on relapse-free and overall survival (**A**) Relapse-free and (**B**) overall survival of patients according to number of *FLT3*-ITD clones detected by HTAS with cDNA template (*n =* 242; one *FLT3*-ITD per patient (*n =* 191), more than one *FLT3*-ITD per patient (*n =* 51)). (**C**) Relapse-free and (**D**) overall survival of patients according to number of *FLT3*-ITD clones detected by fragment analysis with cDNA template (*n =* 242; one *FLT3*-ITD per patient (*n =* 212), more than one *FLT3*-ITD per patient (*n =* 30). HTAS (high-throughput amplicon sequencing), ITD (internal tandem duplication).

**Figure 7:**
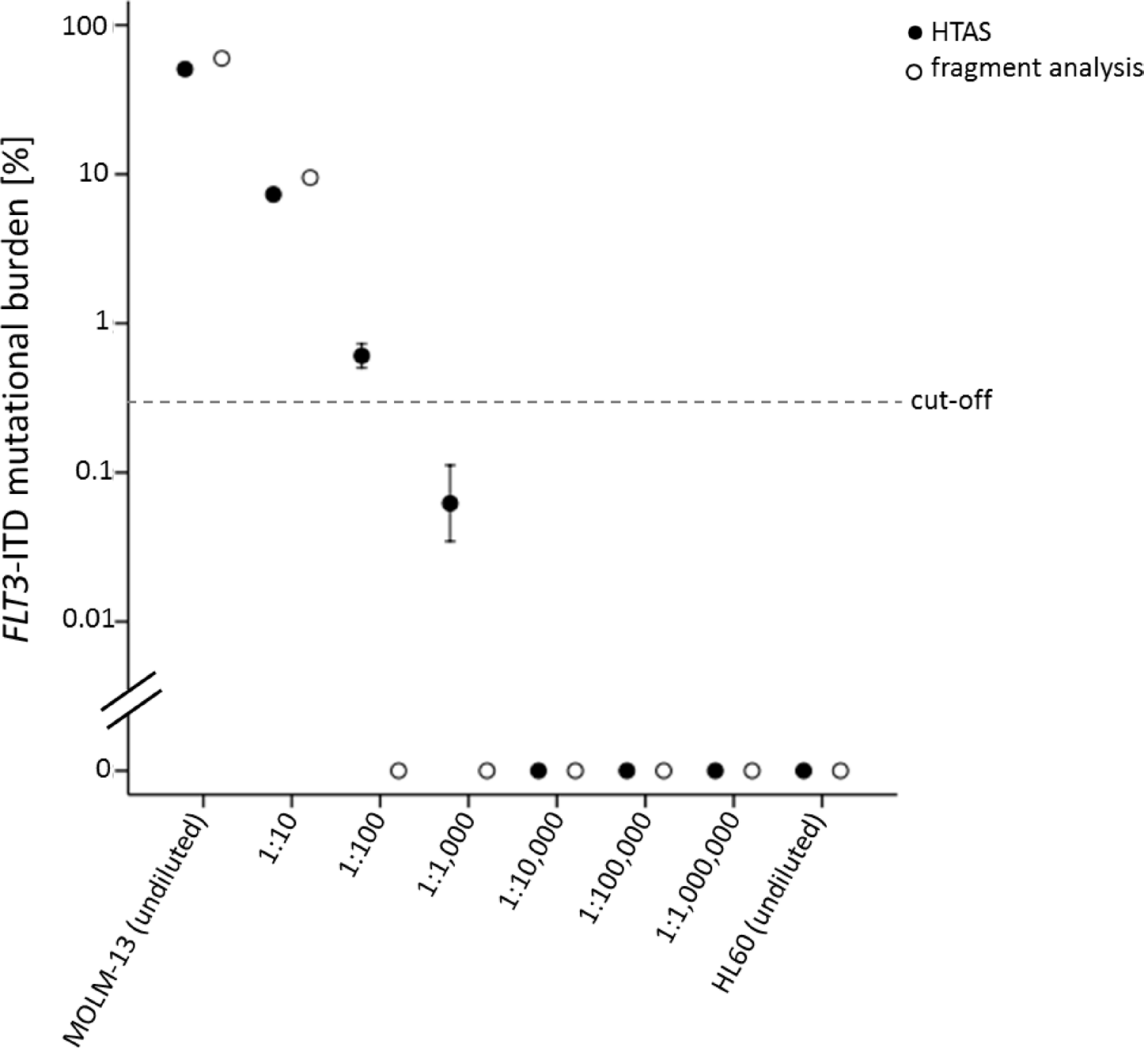
Sensitivity of *FLT3*-ITD detection by HTAS compared to fragment analysis *FLT3*-ITD from the heterozygous *FLT3*-ITD positive cell line MOLM-13 detected after serial dilution in the *FLT3*-WT cell line HL60 by HTAS or fragment analysis using cDNA (*n =* 3; log(10); 95% confidence interval). Cell line cDNA was derived from five million cells each. For amplification 1 µL cDNA template of each serial dilution was used. The dashed line represents the cut-off defined for ITD-analysis in patient samples. HTAS (high-throughput amplicon sequencing), ITD (internal tandem duplication).

For our comparative analysis we selected 250 AML patient samples obtained at initial diagnosis, all *FLT3*-ITD positive according to routine diagnostics (Figure 1), as well as 17 *FLT3*-ITD negative AML samples. All patients were treated on AMLCG trials (AMLCG 1999 [35], AMLCG 2004 [36] or AMLCG 2008 (ClinicalTrials.gov identifier: NCT01382147)) and received an intensive, high-dose cytarabine based induction therapy. The study cohort included patients with all cytogenetic aberrations and was not restricted to patients under the age of 60 years (Table 1). Performing HTAS, we sequenced the same *FLT3-*ITD mutational hot-spot region as covered by cDNA fragment analysis using identical primer sites in both assays (Figure 2 and Table 2). The output of both methods is shown by an exemplary patient in Figure 3. Overall, HTAS detected a total of 312 *FLT3*-ITDs in 242 of 250 *FLT3*-ITD positive patients (97%), compared to 274 ITDs detected by fragment analysis (Supplementary Table 1). The median length of ITD measured by HTAS was 51 nt (range:12–175 nt) at a median allele frequency of 12.2% (range: 0.5–91.1%). By fragment analysis, the median ITD length was 54 nt (range: 15–153 nt) at a median *FLT3*-ITD mRNA level of 0.40 (range: 0.01–0.96). In 11 patients, we observed differences in length between HTAS and routine assays (fragment analysis or Sanger sequencing using cDNA template) with a median variation in length of 6 nt (range: 1–45 nt; median ITD size: 66 nt, range: 12–90 nt; *n =* 11/242; 5%). Validation of the insertion length on the genomic level by targeted sequencing and/or fragment analysis of amplified gDNA confirmed either the results from HTAS or diagnostic routine, each in about half of the cases (Supplementary Table 2). In paired samples (HTAS and Sanger sequencing, *n =* 182), we found identical insertion sites of the dominant ITD in all patients. The highest number of *FLT3*-ITDs was detected in the zipper motif of the JM, followed by the β1-sheet of the TKD1 and then the hinge region of the JM (Figure 4). Neither the insertion site nor the length of ITDs showed any significant correlations with clinical outcome (OS, RFS, and CR rate; Supplementary Tables 3 and 4). In contrast to other studies [16, 37], patients with ITDs in the TKD1 did not show worse clinical outcome compared to patients with ITDs in the JM domain (Supplementary Figure 1). Using HTAS, one ITD was detected in 190 (78.5%) patients, two ITDs were detected in 40 patients (16.5%), three ITDs were detected in nine patients (3.75%) and four ITDs were detected in three patients (1.2%; Figure 5A). In eight patients, who were tested *FLT3*-ITD positive in diagnostic routine, no ITD was detected by HTAS at a variant allele frequency (VAF) above the detection limit (0.5%). However, in four of these eight patients, HTAS detected an ITD consistent with routine results regarding length and position at a VAF below the cut-off. According to routine, another two of these eight patients had each a deletion (three and ten nucleotides) neighbouring or within the ITD. For the remaining two patients, no information about the *FLT3*-ITD mutational burden (*FLT3*-ITD mRNA level) was available from routine diagnostics. Furthermore, HTAS missed eight subclonal ITDs each in one patient reported by routine diagnostics, including three with a length > 75 nt (median *FLT3*-ITD mRNA level: 0.09, range: 0.06–0.25), while 46 additional subclonal ITDs were detected in 34 patients by HTAS only (median VAF: 1.79%, range: 0.50–19.21%; median ITD-supporting reads: 1759, range: 460–23878). The distribution of ITDs over FLT3 domains was the same in HTAS and Sanger sequencing with regards to the dominant clone (Supplementary Figure 2). Out of the 46 additional *FLT3-*ITD clones by HTAS 38 (83%) were validated, ten of which displayed differences in length compared to the genomic level (targeted sequencing and/or fragment analysis with gDNA template; Supplementary Table 5). Overall, HTAS detected more ITDs per patient (mean: 1.27, range: 1–4) compared to fragment analysis (mean: 1.14, range: 1–3; Figure 5B). In contrast to fragment analysis, HTAS revealed a significantly shorter OS and RFS for patients with more than one ITD (HTAS: *p*-value (OS): 0.038, *p*-value (RFS): 0.042; fragment analysis: *p*-value (OS): 0.230, *p*-value (RFS): 0.157; compare Figure 6A and 6B versus 6C and 6D). However, the CR rate did not show any obvious correlation with the number of detected ITDs (data not shown). A multivariate analysis, including *NPM1* mutation status, karyotype and number of *FLT3*-ITD mutations per patient (single versus multiple), did not show significant correlations with clinical outcome (Supplementary Table 6). However, there was a trend for longer RFS associated with single *FLT3*-ITD mutations detected by HTAS (*p*-value: 0.057). A serial dilution of cDNA derived from the heterozygous *FLT3*-ITD positive cell line MOLM-13 in cDNA derived from the *FLT3-*WT cell line HL60 analysed by both methods confirmed higher sensitivity of HTAS (10^–3^, ITD-supporting reads: 58–73 with a coverage of 79,376× to 93,019×) in a 96 sample-setting as compared to fragment analysis (10^−1^, Figure 7). Out of 17 control patients which were *FLT3*-ITD negative according to routine diagnostics, HTAS detected a very small subclonal ITD (VAF: 0.58%) in one patient (UPN C-1; Supplementary Table 1). This subclone could not be validated by gDNA-based fragment analysis (Supplementary Table 5).

**Table 1:** Patient characteristics

Characteristics	
**cohort size** (patients no.)	250
**median age** (years; range)	59; 18–80
**sex (m/f)** (patients no.; [%])	121/129; 48/52
**median follow-up** (months; range)	50; 0–136
**median overall survival** (months; range)	9; 0–136
**Morphologic parameters**	
**median WBC count** (*n* = 243), (leucocytes/mL; range)	48,350; 100–391,200
**median BM-blasts** (*n* = 229), ([%]; range)	83; 10–100
**median PLT** (*n* = 243), (PLT/mL; range)	55,000; 1,220–592,000
**median LDH** (*n* = 239), (LDH/mL; range)	691; 87–6,251
**AML type** (*n* = 245)	**patients** (no.)
***de novo***	205
**s-AML**	27
**t-AML**	13
**FAB-type** (*n* = 236)	**patients** (no.)
**M0**	9
**M1**	71
**M2**	60
**M3**	0
**M4**	67
**M5**	24
**M6**	4
**M7**	1
**Categories according to ELN** (*n* = 246)	**patients** (no.; [%])
**favourable**	0; 0
**intermediate-I**	172; 70
**intermediate-II**	52; 21
**adverse**	22; 9
**Karyotype** (*n* = 250)	**patients** (no.; [%])
**Normal**	176; 70
**Complex**	74; 30
**Molecular genetics**	**patients** (no.; [%])
***FLT3*-ITD+** (*n* = 250)	250; 100
***NPM1*+** (*n* = 189)	118; 47
***CEBPA*+** (*n* = 116)	9; 4
***KMT2A*-PTD+** (*n* = 241)	20; 8

WBC (white blood cell), PLT (platelet counts), BM (bone marrow), LDH (lactate dehydrogenase), AML (acute myeloid leukemia), MDS (myelodysplastic syndrome), ELN (European Leukemia Net), FAB (French American British Classification), ITD (internal tandem duplication), PTD (partial tandem duplication), no. (number).

**Table 2:** *FLT3* primers

Primer for fragment analysis of *FLT3*		
forward		reverse
**cDNA**	5′-FAM-tgt cga gca gta ctc taa aca tg-3′ (R5)	5′-atc cta gta cct tcc caa act c-3′ (R6)
**gDNA**	5′-FAM-gca aat tag gta tga aag cca gc-3′ (11F)	5′-cct tca gca ttt tga cgg caa cc-3′ (12R)

Primer for high-throughput amplicon sequencing of *FLT3*	
forward	reverse
5′-aat gat acg gcg aac aac gag atc tac act ctt tcc cta cac gac gct ctt ccg atc t – BARCODE– tgt cga gca gta ctc taa aca tg -3′	5′ - caa gca gaa gac ggc ata cga gat cgg tct cgg cat tcc tgc tga acc gct ctt ccg atc t – BARCODE – atc cta gta cct tcc caa act c – 3′

FAM (fluorescein amidite).

### HTAS reliably detects small and intermediate insertions but underestimates the mutational burden of long *FLT3*-ITDs

**Figure 8:**
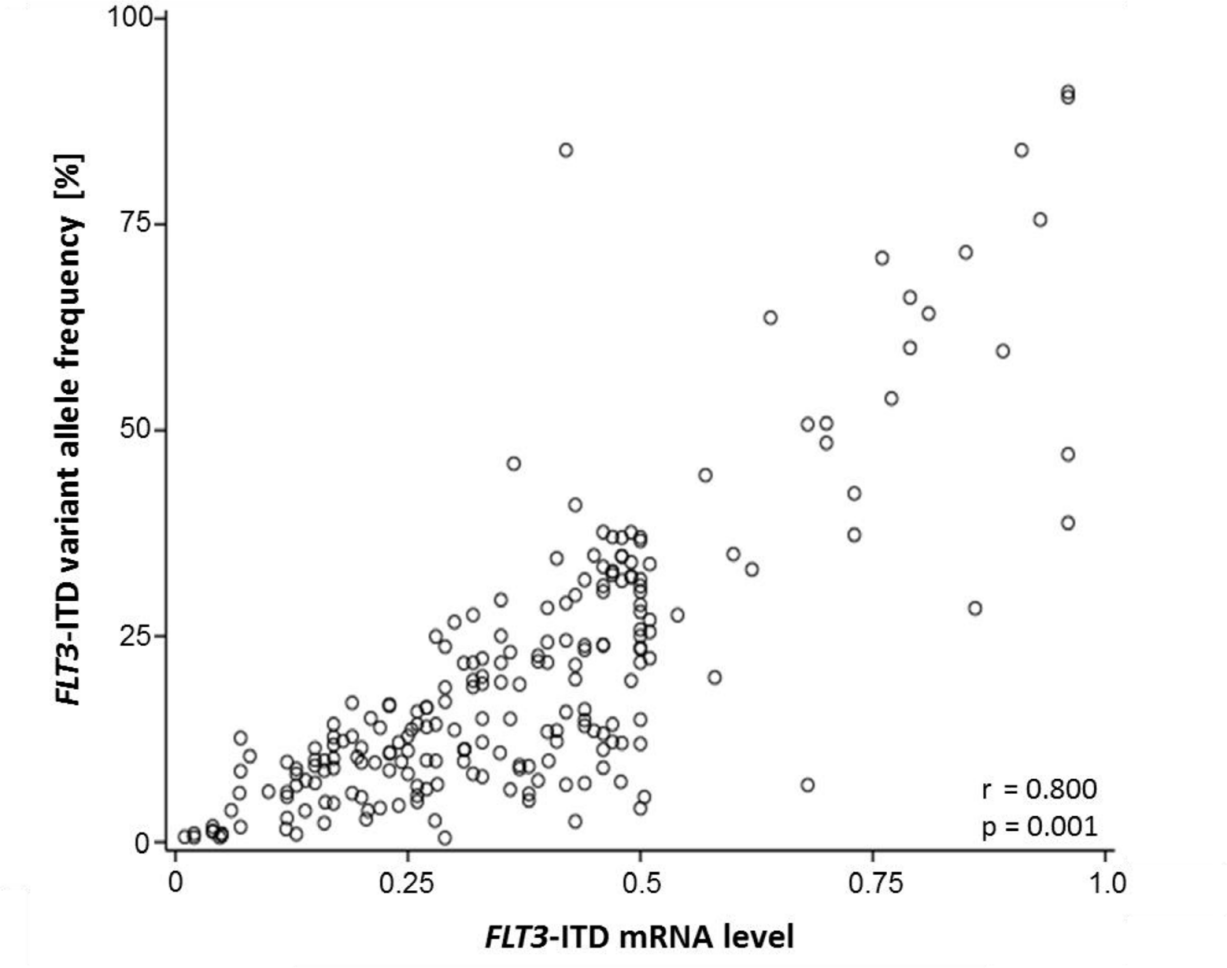
*FLT3*-ITD mutational burden measured by fragment analysis and HTAS Correlation of the *FLT3*-ITD mRNA level according to fragment analysis or according to the variant allele frequency in HTAS using cDNA template (*n =* 220). HTAS (high-throughput amplicon sequencing), ITD (internal tandem duplication).

The VAF in HTAS showed a strong correlation with the mutational burden detected by fragment analysis (Pearson: 0.758, *p*-value: 0.001, *n =* 220; Figure 8). Consistent with previous reports [26, 38], high *FLT3*-ITD mRNA levels (>0.5) measured by fragment analysis showed a significant correlation with shorter relapse-free survival (RFS) and overall survival (OS) (Supplementary Figure 3A and 3B). This correlation could also be observed for *FLT3*-ITD levels by HTAS for RFS and as trend for OS (Supplementary Figure 3C and 3D). Given that the *FLT3*-ITD/*FLT3*-WT ratio based on the sum of all ITD clones is recommended by the ELN for standard of care [8], we calculated the total *FLT3*-ITD mutational burden per patient. Adding the mutational *FLT3*-ITD burden of ITD subclones increased the significance of the correlations between ITD load and outcome (Supplementary Figure 4A–4C). This was also evident in multivariate analysis (Supplementary Table 6). A total *FLT3*-ITD mRNA load below 50% was an independent favorable prognostic factor for outcome measured by fragment analysis for both RFS (*p*-value: 0.005) and OS (*p*-value: 0.020). For HTAS a low ITD mutation load correlated significantly with longer RFS in multivariate analysis (*p*-value: 0.025).

**Figure 9:**
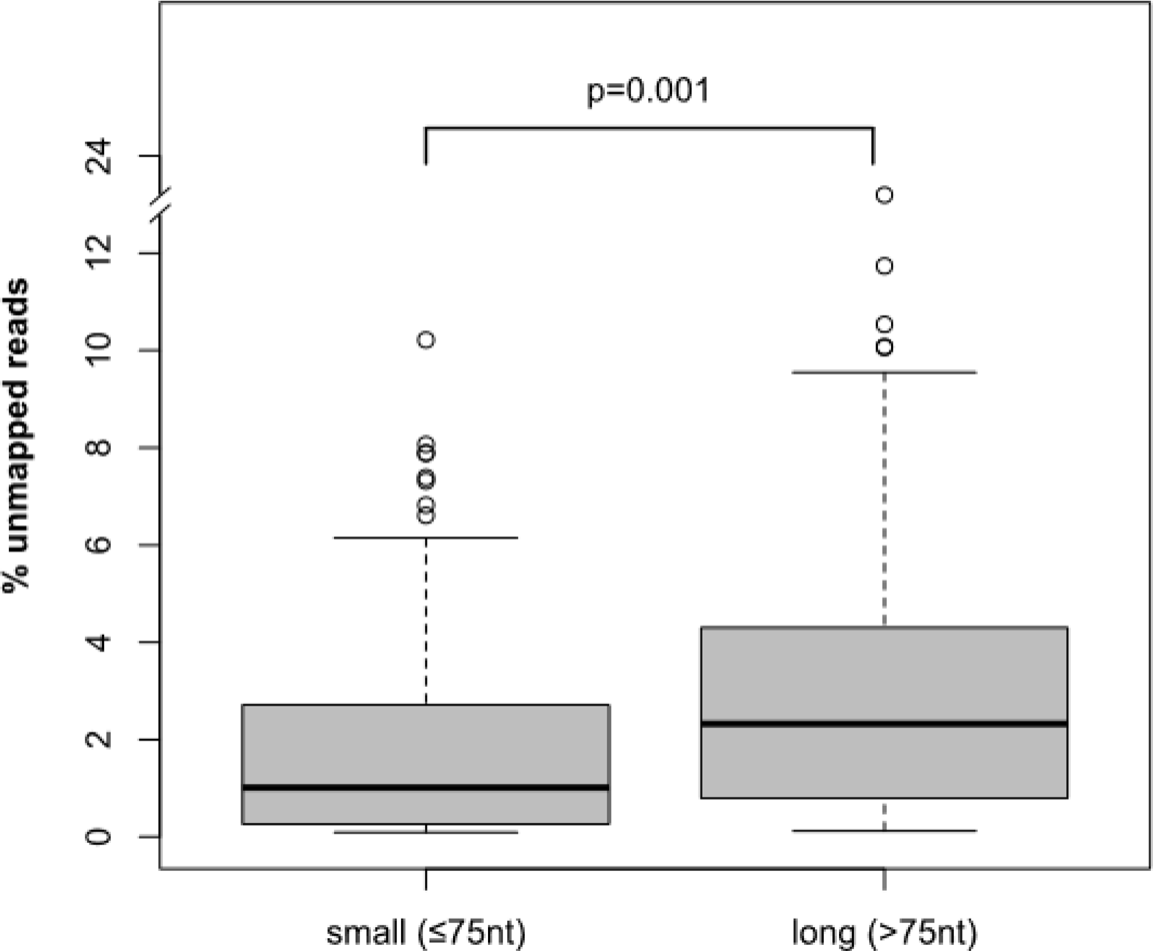
Mappability of *FLT3*-ITD sequence reads Mappability of sequence reads from HTAS according to length of ITD. *P*-value was computed using Mann-Whitney-*U* test. nt (nucleotide), HTAS (high-throughput amplicon sequencing), ITD (internal tandem duplication).

The VAF levels of *FLT3*-ITD measured by HTAS were up to 5-times lower than the *FLT3*-ITD mRNA level measured by fragment analysis. Interestingly, the difference in mutational burden between HTAS and fragment analysis increased with ITD length irrespective of clonal dominance (Spearman: 0.530, *p*-value: 0.001, *n =* 220; Supplementary Figure 5A and 5B). Using HTAS, long ITDs were detected on average with lower VAF compared to short ITDs (Supplementary Figure 5C, Spearman: −0.249, *p*-value: 0.001, *n =* 312), while fragment analysis measured *FLT3*-ITD mRNA levels more accurately, regardless of ITD length, with only a minor decrease for long ITDs (Supplementary Figure 5D; Spearman: –0.054, *p*-value: 0.418, *n =* 228). In HTAS, the number of ITD-supporting reads was negatively correlated with ITD length, while the total number of reads was similar in short and long ITDs (Pearson: 0.309, *p*-value: 0.001, *n =* 242). This correlation is likely due to the fact that long ITDs were more difficult to map to the reference sequence. Samples harbouring ITDs with a length < 75 nt showed significantly fewer unmapped reads compared to samples harbouring ITDs with a length >75 nt (Mann-Whitney-*U* test, *p*-value: <0.001; Figure 9). The ITD position was also related to the difference in mutational burden between HTAS and fragment analysis (Supplementary Figure 6), with longer insertions at cDNA nucleotide positions encoding C-terminal domains of FLT3 (Spearman: 0.536, *p*-value: 0.001, *n =* 312; Supplementary Figure 7). Validation of ITDs using gDNA in 43 patient samples furthermore revealed differences in the *FLT3*-ITD levels from those measured using cDNA in several cases. Besides the underestimation of the mutational burden for long ITDs by HTAS, discrepancies might be attributed to transcriptional imbalance favouring either the WT or the ITD allele. Overall and in line with published data [23, 39], *FLT3*-ITD levels measured by HTAS with cDNA template showed a strong correlation with the genomic levels measured by fragment analysis (Spearman: 0.846, *p*-value: <0.001, *n =* 86 ITDs; Supplementary Figure 8A) and targeted haloplex sequencing (Spearman: 0.752, *p*-value: <0.001, *n =* 41 ITDs, Supplementary Figure 8B). Comparison of genomic and transcriptional *FLT3*-ITD levels measured by fragment analysis excluded gross methodological differences. However, the distribution pointed towards a transcriptional imbalance in favour of the ITD allele, consistent with a moderate correlation (Spearmann: 0.554, *p*-value: <0.001, *n =* 40 ITDs, Supplementary Figure 8C). Internal cell line controls were sequenced in each of the four instrument runs. The *FLT3*-ITD positive cell line MOLM-13 displayed the expected *FLT3*-ITD (size: 21 nt; cDNA position: 1774) [40]. Comparison of the mutational burden in the heterozygous *FLT3*-ITD positive cell line MOLM-13 between instrument runs revealed a lower experimental variance in HTAS (0.17%, mean VAF ± standard deviation: 49.24 ± 0.36) compared to fragment analysis (4.92%, mean *FLT3*-ITD mRNA level ± standard deviation: 0.49 ± 0.02; Supplementary Figure 9), indicating high inter-run reproducibility and accuracy of HTAS.

### *FLT3*-ITD detection by HTAS requires controls to exclude ITD artifacts

We detected ITD artifacts, which were present in the negative control cell line HL60 and the *FLT3*-ITD negative patient samples, at VAF levels below our cut-off of 0.5% (Supplementary Table 7). By manual inspection, these two ITD artifacts were therefore excluded from the analysis, even if occurring in patient samples at VAFs above the cut-off of 0.5%. This applied for the artifact ITD at cDNA position 1712 in 148 (61%) of analysed samples (median VAF: 0.99%; range: 0.5–4.71%). Furthermore, we identified and excluded a second ITD artifact within the primer region (cDNA position: 1831–1848), occurring at reference cDNA position 1837 exclusively. This ITD artifact occurred as subclone in 36 patients (15%), at a median VAF of 0.93% (range: 0.5–3.71%) and a median size of 68 nt (range: 35–107 nt). These ITD artifacts showed high sequence similarity to the ITD of the predominant clone, however, they were one nt shorter and displayed 1/20 of the VAF of the original *FLT3*-ITD (median length: 69 nt, range: 36–108 nt; median VAF: 20.57%, range: 6.38–79.49%, example shown in Supplementary Table 7). Thus, this points towards a PCR-based mispriming event, similar to those reported for false-positive calls in multiplex PCR-based NGS approaches [41].

### Mutations in other genes do not correlate with ITD position, length or clonality

Individual patients were evaluated for mutations in *NPM1*, *CEBPA*, *KIT*, *IDH1/2* and *KMT2A-*PTD. One hundred nineteen of 182 patients (65%) were positive for an *NPM1* mutation, nine out of 112 patients (8%) were positive for a *CEBPA* mutation (three *CEBPA*-double positive), two out of 164 (1%) were positive for a *KIT* mutation, 14 out of 75 (19%) were positive for a *IDH1/2* mutation, 19 out of 233 patients (8%) were positive for a *KMT2A-*PTD mutation. A point mutation in the TKD of *FLT3* was found in six out of 208 patients (3%). Other mutations, in amongst others *NRAS* and *RUNX1*, occurred in up to four patients per affected gene. There was no correlation of *FLT3*-ITD clonality with other co-occurring mutations (Supplementary Figure 10). Mutations were equally distributed between the patients when clustered according to FLT3 domains (Table 3). Neither length nor position of *FLT3*-ITD correlated with the co-occurrence of other mutations. In agreement with published results [42], *NPM1* mutations correlated with better clinical outcome in our study cohort (Supplementary Figure 11). Patients positive for *FLT3*-ITD and *NPM1* mutation had a significantly increased RFS compared to *FLT3*-ITD positive and *NPM1* negative patients (*p*-value: 0.049). For OS, a similar trend with borderline significance was observed. In line with published results [35, 43–45], an *NPM1* mutation remained an independent favorable prognostic factor in multivariate analysis (Supplementary Table 6). In contrast, the other evaluated mutations did not show any correlations with clinical outcome in our *FLT3*-ITD positive study cohort.

**Table 3:** *FLT3*-ITD localization and co-occurrence of mutations in other cancer related genes

mutation	juxtamembrane domain			tyrosine kinase domain			total
	switch motif	zipper motif	hinge region		beta1-sheet	NBL	
***FLT3-*PM (TKD)**	0	0(2)	3		2	1(1)	6(3)
***NPM1***	3	58	18		29	7	115
***CEBPA***	0	4	1		3	1	9
***KIT***	0	1	1		0	0	2
***IDH1/2***	0	7	2		5	0	14
***KMT2A-*PTD**	0	10	2		6	1	19
***FLT3-*ITDs**	4	128	35		62	13	242

Distribution of concurrent mutations among the patients, clustered according to ITD location in functional FLT3 domain (*n =* 242, according to *FLT3*-ITD position of the dominant clone by high-throughput amplicon sequencing (HTAS)). PM (point mutations; including D835N and V592L), ITD (internal tandem duplication), TKD (tyrosine kinase domain), NBL (nuclear binding loop), PTD (partial tandem duplication), no. (number).

## DISCUSSION

In this study, we report the comparison of two different methods to detect *FLT3*-ITD mutations in 250 adult *FLT3*-ITD positive AML patients. Since *FLT3*-ITD parameters, including position and mutational burden, are of clinical relevance for risk stratification and therapy decision [1, 6, 9, 10, 16, 26, 46], a fast and reliable detection method for routine diagnostics is essential. Therefore, we established a high-throughput *FLT3*-ITD amplicon sequencing assay to gain information complementary to the results from routine fragment analysis. Although HTAS identified nearly all dominant ITD clones that were detected by routine diagnostics, we encountered technical limitations consistent with previous studies investigating smaller cohorts [47, 48]. In particular, we found methodological differences between HTAS and fragment analysis with respect to ITD length and the quantification of the *FLT3*-ITD mutational burden. In 5% of our patients the ITD length differed when comparing HTAS and fragment analysis results. In contrast to other studies with lower patient numbers [16, 22, 29], the ITD length and position did not correlate with clinical outcome in our study. Concordant with other reports [16, 17, 49, 50], the presence of ITDs with high mutational burden correlated with worse prognosis. In multivariate analysis, the *FLT3*-ITD level below 50% was an independent favorable prognostic factor. In our study, HTAS revealed more subclonal ITDs compared to fragment analysis and the additional clonal complexity uncovered by HTAS correlated with adverse clinical outcome. In line with our findings, it was recently shown that the number of driver mutations has prognostic relevance in MDS and AML [3, 51, 52]. It has already been suggested by others to consider the number of *FLT3*-ITD clones per patient for prognostic stratification [38, 49, 53]. Whereas in our analysis the *FLT3*-ITD clonality (single versus multiple) did not reach statistical significance in multivariate analysis, we observed a trend for better outcome associated with single alterations when evaluated by HTAS. Although the scalable sensitivity of NGS approaches based on read depth seems attractive, the sensitivity might be limited by false-positive variant calls as observed in the present study. Therefore, the implementation of appropriate negative controls is essential. Moreover, NGS data has to be analyzed carefully to identify and to exclude ITD artifacts as detected at cDNA position 1837 in more than 15% of our patients.

Besides clonalilty, another important prognostic parameter is the *FLT3*-ITD mutational burden. Although there was a significant correlation between *FLT3*-ITD mRNA level detected by fragment analysis and variant allele frequency by HTAS, the prognostic relevance of *FLT3*-ITD mutational burden was more pronounced when considering the results from fragment analysis. Of note, in fragment analysis the *FLT3*-ITD mRNA levels were up to five-fold higher for patient samples compared to the VAF determined by HTAS. Consistent with results from a study utilizing Pindel software as well as a custom *de-novo* assembly approach for *FLT3*-ITD detection [54], we found that HTAS underestimated the *FLT3*-ITD mRNA levels of long ITDs, which are less likely to be mapped correctly compared to shorter ITDs. In our study, ITDs with a length > 75 nt showed inappropriate mapping to the reference sequence. Interestingly, ITD detection by Pindel was recently shown to be dependent on the length and on the relative position of the amplicon [47]. Thus, algorithms for read mapping and variant detection are currently limiting the detection of long ITDs. This limitation might be overcome with increasing read length, as in the present study 2 × 250 bp paired-end reads did not provide sufficient bi-directional coverage of long ITDs. Since the *FLT3*-ITD/*FLT3*-WT ratio, which is of prognostic value [4, 26, 28, 38, 49, 55], is currently measured more accurately by fragment analysis, ITD quantification by HTAS needs to be further optimized. On the other hand, detection of ITD subclones by NGS-based approaches with increased sensitivity contributes to the total *FLT3*-ITD mutational burden and thus increases the *FLT3*-ITD/*FLT3*-WT ratio. Superior sensitivity of NGS is also relevant for MRD monitoring in leukemia patients. In our serial dilution, HTAS reached a sensitivity of 10^–3^ for the heterozygous *FLT3*-ITD positive cell line MOLM-13, in a multi-sample sequencing setting of 96 samples per run, being superior to fragment analysis. Since sensitivity and coverage is scalable, depending on the number of samples per run, a higher sensitivity can be achieved, if required, as shown by others [30]. Although *FLT3*-ITD is a rather variable marker during therapy and disease progression, with mutational plasticity between diagnosis and relapse [56–59], several studies [27, 30, 33] have pointed out the advantages of NGS approaches for *FLT3*-ITD MRD assessment over conventional diagnostic applications. Especially, the recently approved TKI treatment with Midostaurin in combination with induction chemotherapy for *FLT3*-mutated AML [13] requires the reliable reveillance of *FLT3* mutations for initial risk assessment, monitoring and therapeutic intervention. In a recent study, *FLT3*-mutation positive relapsed or refractory AML CHRYSALIS Phase I/II study patients were analysed for their TKI-response to Gilteritinib (ASP2215; clincal trail number: NCT02014558 [60]). Interestingly, the clinical response correlated with the post-treatment *FLT3*-ITD MRD levels, evaluated by NGS [61]. In addition, the identification of the ITD position by NGS approaches might be of prognostic relevance considering the impact of *FLT3*-ITD insertion site on therapy resistance and outcome – for conventional chemotherapy as well as for TKI treatment [10, 16–21]. However, the clinical relevance of ITD position still remains controversial [22, 24, 25, 38]. Furthermore, newly arising *FLT3* point mutations during clonal evolution, as for example D835Y and D835G, have prognostic impact as being capable of mediating TKI resistance [33]. Thus, reads spanning the *FLT3* regions JM to TKD2 should be used in future approaches as sequence read length steadily increases. With multiple parameters obtained from a single assay and hands-on time for sample preparation and analysis similar to *FLT3*-ITD fragment analysis or Sanger sequencing, NGS techniques become more and more attractive for diagnostic laboratories. Down-scaling of our 96-sample setting according to the needs in diagnostic routine is feasible when using recently introduced scalable sequencing instruments and reagents. HTAS may enable more precise therapy decisions based on the detection of small ITD clones and has a strong pontential for monitoring of FLT3-directed interventions.

In summary, our study demonstrates the feasibility of HTAS for *FLT3*-ITD detection in AML. We show that the sensitive *FLT3*-ITD subclone detection by HTAS is of prognostic relevance and has the potential to shed light on the clonal architecture of AML. However, the detection of long ITDs and the detection of ITDs in combination with deletions remain challenging. Increasing read length as well as improving variant detection algorithms will likely help to overcome these limitations. After methodological improvement, HTAS may serve as a robust and sensitive tool that could be implemented in future diagnostic routines, essential for a rapid risk stratification and therapeutic intervention.

## MATERIALS AND METHODS

### Patient samples

This study included 267 newly diagnosed patients with AML, of which 250 were *FLT3*-ITD positive according to routine diagnostics (median age: 59, range: 18–80 years; Figure 1). All patients were treated intensively according to the German Acute Myeloid Leukemia Cooperative Group (AMLCG) clinical trial study protocols with curative intent (AMLCG 1999 (ClinicalTrials.gov identifier: NCT00266136) and 2004 as published [35, 36], AMLCG 2008 (*n =* 38, ClinicalTrials.gov identifier: NCT01382147)), which were approved by the institutional review boards of the participating centers. Patients with acute promyelocytic leukemia (FAB M3) were not treated on these trials, and therefore not analysed in our study. Informed written consent was obtained from all patients in accordance with the Declaration of Helsinki. According to the ELN-classification [9] patients clustered into the following groups: intermediate I (70%), intermediate II (21%) or adverse (9%). A normal karyotype was observed in 70%, while 30% had a complex aberrant karyotype. After intensive induction chemotherapy, 142 (56.8%) patients achieved CR, 16 (6.6%) patients achieved CRi, while 34 (13.6%) patients had refractory disease, 35 (14.0%) patients died during induction therapy, and for 23 (9.1%) patients no remission status was available. Hematopoietic stem cell transplantation in first CR was performed in 36 (14.4%) patients. After a median follow-up of 50 months (range: 0–136), 97 (68.3%) patients that had reached CR eventually relapsed and 187 (74.8%) of all patients died. The median RFS and OS observed were seven and nine months, respectively. The two-year rates of RFS and OS were 20.0% and 25.2%, respectively. Samples were collected at the University Hospital LMU Munich at the time point of first diagnosis. Mononuclear cells were isolated from bone marrow aspirates or peripheral blood and subjected to routine diagnostics for conventional cytogenetic and routine mutational analysis of known molecular markers, including *NPM1, CEBPA* and *FLT3*-ITD, according to standard protocols [2, 26, 35, 62]. Patient characteristics and clinical parameters are provided in Table 1.

### Cell lines

All cell lines were purchased from the German Collection of Microorganisms and Cell Culture (DSMZ, Braunschweig, Germany), with certified cell authentication using karyotyping and fluorescent *in-situ* hybridization, immunophenotyping, and testing of cancer-type specific mutations using RT-PCR analyses [63, 64]. AML cell lines positive for *FLT3*-ITD (MOLM-13, ACC-554) and negative for *FLT3*-ITD (HL60, ACC-3) were cultivated according to the supplier’s recommendations. Cell lines were tested for a mycoplasma contamination on a regular basis (MycoAlert Mycoplasma Detection Kit, Lonza Rockland Inc., Rockland, ME, USA).

### DNA and RNA isolation

Extraction of DNA and RNA was performed using standard procedures. Per sample five million cells were used for DNA or RNA isolation each. gDNA and total RNA were extracted using the QIAamp DNA Mini Kit (51106, Qiagen) or RNeasy Mini Kit (74106, Qiagen, Hilden, Germany), respectively, utilizing a QIAcube (Qiagen) according to the suppliers’ recommendations. Purity of isolated RNA and DNA was verified by absorbance measurements (260/280 ratios) with a Nanodrop instrument (Peqlab Biotechnology, Erlangen, Germany).

### cDNA synthesis

cDNA synthesis was performed by reverse transcription (RT) using total RNA extracted out of five million cells, the Superscript II Reverse Transciptase and corresponding buffer (18064071, Invitrogen – ThermoFisher Scientific, Munich, Germany), 100 mM dNTPs Set (10297-117, Invitrogen–ThermoFisher Sicientific), 25 µM Random Primer p(ND)6 (1034731, Roche Diagnostics, Penzberg, Germany) and RNAse Inhibitor (N2615, Promega, Mannheim, Germany). Reactions were performed on a thermocycler (Peqlab Biotechnology), according to the technical protocol of the Superscript II Reverse Transciptase (70° C 10 min, 37° C 120 min and 90° C 5 min, 1 cycle each).

### *FLT3*-ITD fragment analysis

For fragment analysis, PCR (amplification for 28 cycles –1 min 95° C, 1 min 60° C, 1 min 72° C) with 1 µL patient or cell line cDNA or gDNA (10 ng/µL) template, respectively, was performed in a 25 µL reaction volume to amplify *FLT3*, utilizing fluorescently-labelled primers (10 pmol each, Table 2, Metabion, Planegg, Germany) as published [65, 66] and the *Taq* PCR Master Mix (201445, Qiagen). Thereafter, 0.5 µL fragment-length standard (GeneScan (500) ROX Size Standard, 401734, Applied Biosystems, Foster City CA, USA) and 13.5 µL PCR-grade water were added to 1 µL PCR-product. After initial denaturation of this mixture at 95° C, size-separation by capillary electrophoresis was performed on a Genetic Analyzer 3500xl (Applied Biosystems) using the separation matrix POP-6 polymer (4316357, Applied Biosystems). Data analysis was performed using the Gene-Mapper software (Version 3.5, Thermo Fisher Scientific). *FLT3*-ITD mutational burden (*FLT3*-ITD mRNA level respectively) was calculated based on the WT to ITD ratio equation as previously published [26]. Comparative analysis of detected ITDs was based on cDNA fragment analysis, whilst gDNA fragment analysis, referred to as g(F), was performed as validation.

### *FLT3* amplification and Sanger sequencing

For Sanger sequencing PCR (amplification for 35 cycles – 1 min 95° C, 1 min 60° C, 1 min 72° C) with 1 µL patient cDNA template was performed in a 25 µL reaction volume to amplify *FLT3,* using the *FLT3* primers 11F and 12R (10 pmol each, sequence as described [65]) and *Taq* PCR Master Mix (201445, Qiagen). After PCR-product purification with the Qiaquick PCR Purifcation Kit (28106, Qiagen), bi-directional sequencing was performed with a second round of amplification using the Big Dye Terminator v1.1 kit (4337451, Life Technologies) and the *FLT3* primer 11F and 12R (10 pmol each, sequence as described [65]). Samples were purified using the CentriSep 8 solution and Columns (CS-912, Princeton Separations) and subsequently sequenced using a Genetic Analyzer 3500xl (Applied Biosystems) according to the manufacturers’ instructions. Chromatograms were analyzed using Sequencher Software 5.1 (Gene Codes Cooperation, MI, USA) and the *FLT3* cDNA reference sequence (NM_004119.2).

### High-throughput *FLT3-*ITD amplicon sequencing (HTAS) and genomic targeted sequencing

In case of HTAS, *FLT3* amplicons were generated performing PCR (TD58, amplification for 30 cycles – 30 sec 95° C, 30 sec 58° C, 1 min 72° C) with 1 µL cDNA template in a 25 µL reaction volume, using the *Taq* PCR Master Mix Kit (201445, Qiagen) and custom-designed *FLT3* cDNA primers (10 pmol each, Table 2). The *FLT3* cDNA primers, spanning the mutational hotspot region (spanning 366 base-pairs (bp)), included a barcode and Illumina-specific adapter-sequences (Supplementary Table 8), enabling a one-step PCR-protocol for barcoded *FLT3*-targeted amplification and multiplex-sequencing. As controls, cDNA from *FLT3*-ITD positive (MOLM-13) and negative (HL60) human cell lines were amplified. Correct amplicon fragment size was verified by agarose gel-electrophoresis. The PCR-product was purified utilizing NucleoFast 96 PCR clean-up plates (743100, Machery-Nagel, Düren, Germany) according to the manufacturers’ instructions. DNA concentration was measured using the Quant-iT dsDNA Broad Range High Sensitivity Kit (Q33120, Thermo Fisher Scientific) in compliance with the manufacturers’ protocol, following dilution to a final concentration of 4 nM (4 fmol/µL). Library preparation was performed according to the manufacturers’ instructions using the MiSeq Kit v2 500 cycles (MS-102–2003, Illumina, San Diego CA, USA), while adding PhiX control (PhiX Control v3 Kit, FC-110-3001, Illumina) in a ratio 4:1 (800 µL library, 200 µL PhiX control). Per library, up to 96 samples were pooled, adding 10 µL of each 4 nM sample. The cell lines HL60 and MOLM-13 were included in every instrument run, serving as inter-run *FLT3*-WT and *FLT3*-ITD control. Sequencing (2 × 250 bp paired-end) was performed on a MiSeq Personal Sequencer instrument (Illumina, San Diego CA, USA) in four independent runs, yielding a median of 79,110 reads per amplicon (range: 31,996–259,783). For validation purposes genomic targeted sequencing data, was obtained from a previous study, generated by utilization of an HaloPlex target enrichment system (Agilent Technologies, Santa Clara, USA) as described previously [3].

### Next generation sequencing data analysis

Raw sequence reads were aligned to the *FLT3* cDNA reference sequence (NM_004119.2) using BWA [67]. Parameters were adjusted in order to allow the incorporation of long insertions, i.e. minimized gap open and gap extension penalties, maximum number of gap extensions. Analysis of NGS-data was performed using the Galaxy platform [68, 69]. Tandem duplications were called using Pindel (version 0.2.5a7) [70] with a minimum size of 6 bp and a minimum of 10 supporting reads. Pindel reports left-aligned positions of variants, which is the leftmost possible position of an alteration. As tandem repeats are two identical and consecutive sequences, Pindel reports the starting position of the first sequence (i.e. the 5′-template) by default. In order to indicate the position of the inserted sequence (i.e. the 3′-duplicate), we added the length of the ITD to the position reported by Pindel. Furthermore, the 5′-UTR region (82 nucleotides), included in the *FLT3* cDNA reference sequence, were substracted from the variant position. Thus, results from HTAS could be compared to results from Sanger sequencing, which refer to the coding sequence. The VAF was computed by dividing the number of supporting reads with the coverage at the insertion site. Minimum cut-off for VAF was set to 0.5% (HTAS), based on empirical analysis of *FLT3*-ITD negative control samples, to exclude any sequencing background noise (non-specific variants). Off-target ITDs and ITDs which appeared in the *FLT3*-WT cell line HL60 were excluded from further analysis. Per sample the median coverage per amplicon was 157,100× (range: 63,890× – 488,300×).

### Statistical analysis

SPSS (IBM, version 21.0) or R (GNU GPL, version 3.4.1) software was used for statistical analysis. Spearman rank correlation was used to examine correlations between continuous parameters. Two-sided log rank test was used for Kaplan-Meier diagrams to compute survival curves. Mann-Whitney-*U* test was performed to test for differences between groups. Results were considered as significant with a *p*-value less than 0.05. Univariate and multivariate Cox-Regression analysis was performed to evaluate prognostic variables for OS and RFS. RFS was calculated from the time span between diagnosis and relapse [9, 71], failure to achieve a complete remission (CR) [35], last follow-up or death. OS was evaluated from initial diagnosis to last follow up or death. The criteria of relapse, resistant disease and CR were assigned according to ELN [9]. Cox proportional hazard model was used to estimate hazard ratios for multivariate analysis.

## SUPPLEMENTARY MATERIALS FIGURES AND TABLES





## References

[1] Mrozek K, Marcucci G, Paschka P, Whitman SP, Bloomfield CD (2007). Clinical relevance of mutations and gene-expression changes in adult acute myeloid leukemia with normal cytogenetics: are we ready for a prognostically prioritized molecular classification?. Blood.

[2] Schnittger S, Schoch C, Dugas M, Kern W, Staib P, Wuchter C, Loffler H, Sauerland CM, Serve H, Buchner T, Haferlach T, Hiddemann W (2002). Analysis of FLT3 length mutations in 1003 patients with acute myeloid leukemia: correlation to cytogenetics, FAB subtype, and prognosis in the AMLCG study and usefulness as a marker for the detection of minimal residual disease. Blood.

[3] Metzeler KH, Herold T, Rothenberg-Thurley M, Amler S, Sauerland MC, Gorlich D, Schneider S, Konstandin NP, Dufour A, Braundl K, Ksienzyk B, Zellmeier E, Hartmann L (2016). Spectrum and prognostic relevance of driver gene mutations in acute myeloid leukemia. Blood.

[4] Thiede C, Steudel C, Mohr B, Schaich M, Schakel U, Platzbecker U, Wermke M, Bornhauser M, Ritter M, Neubauer A, Ehninger G, Illmer T (2002). Analysis of FLT3-activating mutations in 979 patients with acute myelogenous leukemia: association with FAB subtypes and identification of subgroups with poor prognosis. Blood.

[5] Fröhling S, Schlenk RF, Breitruck J, Benner A, Kreitmeier S, Tobis K, Döhner H, Döhner K, AML Study Group Ulm (2002). Acute myeloid leukemia. Prognostic significance of activating FLT3 mutations in younger adults (16 to 60 years) with acute myeloid leukemia and normal cytogenetics: a study of the AML Study Group Ulm. Blood.

[6] Levis M, Small D (2003). FLT3: ITDoes matter in leukemia. Leukemia.

[7] Levis M (2013). FLT3 mutations in acute myeloid leukemia: what is the best approach in 2013?. Hematology Am Soc Hematol Educ Program.

[8] Dohner H, Estey E, Grimwade D, Amadori S, Appelbaum FR, Buchner T, Dombret H, Ebert BL, Fenaux P, Larson RA, Levine RL, Lo-Coco F, Naoe T (2017). Diagnosis and management of AML in adults: 2017 ELN recommendations from an international expert panel. Blood.

[9] Dohner H, Estey EH, Amadori S, Appelbaum FR, Buchner T, Burnett AK, Dombret H, Fenaux P, Grimwade D, Larson RA, Lo-Coco F, Naoe T, Niederwieser D (2010). Diagnosis and management of acute myeloid leukemia in adults: recommendations from an international expert panel, on behalf of the European LeukemiaNet. Blood.

[10] Schlenk RF, Kayser S, Bullinger L, Kobbe G, Casper J, Ringhoffer M, Held G, Brossart P, Lubbert M, Salih HR, Kindler T, Horst HA, Wulf G (2014). Differential impact of allelic ratio and insertion site in FLT3-ITD-positive AML with respect to allogeneic transplantation. Blood.

[11] Bornhäuser M, Illmer T, Schaich M, Soucek S, Ehninger G, Thiede C, AML SHG 96 study group (2007). Improved outcome after stem-cell transplantation in FLT3/ITD-positive AML. Blood.

[12] Ho AD, Schetelig J, Bochtler T, Schaich M, Schafer-Eckart K, Hanel M, Rosler W, Einsele H, Kaufmann M, Serve H, Berdel WE, Stelljes M, Mayer J (2016). Allogeneic Stem Cell Transplantation Improves Survival in Patients with Acute Myeloid Leukemia Characterized by a High Allelic Ratio of Mutant FLT3-ITD. Biol Blood Marrow Transplant.

[13] Rasko JEJ, Hughes TP (2017). First Approved Kinase Inhibitor for AML. Cell.

[14] Levis M (2017). Midostaurin approved for FLT3-mutated AML. Blood.

[15] Stone RM, Mandrekar SJ, Sanford BL, Laumann K, Geyer S, Bloomfield CD, Thiede C, Prior TW, Dohner K, Marcucci G, Lo-Coco F, Klisovic RB, Wei A (2017). Midostaurin plus Chemotherapy for Acute Myeloid Leukemia with a FLT3 Mutation. N Engl J Med.

[16] Kayser S, Schlenk RF, Londono MC, Breitenbuecher F, Wittke K, Du J, Groner S, Spath D, Krauter J, Ganser A, Dohner H, Fischer T, Dohner K (2009). Insertion of FLT3 internal tandem duplication in the tyrosine kinase domain-1 is associated with resistance to chemotherapy and inferior outcome. Blood.

[17] Schnittger S, Bacher U, Haferlach C, Alpermann T, Kern W, Haferlach T (2012). Diversity of the juxtamembrane and TKD1 mutations (exons 13-15) in the FLT3 gene with regards to mutant load, sequence, length, localization, and correlation with biological data. Genes Chromosomes Cancer.

[18] Breitenbuecher F, Markova B, Kasper S, Carius B, Stauder T, Bohmer FD, Masson K, Ronnstrand L, Huber C, Kindler T, Fischer T (2009). A novel molecular mechanism of primary resistance to FLT3-kinase inhibitors in AML. Blood.

[19] Breitenbuecher F, Schnittger S, Grundler R, Markova B, Carius B, Brecht A, Duyster J, Haferlach T, Huber C, Fischer T (2009). Identification of a novel type of ITD mutations located in nonjuxtamembrane domains of the FLT3 tyrosine kinase receptor. Blood.

[20] Heidel F, Solem FK, Breitenbuecher F, Lipka DB, Kasper S, Thiede MH, Brandts C, Serve H, Roesel J, Giles F, Feldman E, Ehninger G, Schiller GJ (2006). Clinical resistance to the kinase inhibitor PKC412 in acute myeloid leukemia by mutation of Asn-676 in the FLT3 tyrosine kinase domain. Blood.

[21] Fischer M, Schnetzke U, Spies-Weisshart B, Walther M, Fleischmann M, Hilgendorf I, Hochhaus A, Scholl S (2017). Impact of FLT3-ITD diversity on response to induction chemotherapy in patients with acute myeloid leukemia. Haematologica.

[22] Stirewalt DL, Kopecky KJ, Meshinchi S, Engel JH, Pogosova-Agadjanyan EL, Linsley J, Slovak ML, Willman CL, Radich JP (2006). Size of FLT3 internal tandem duplication has prognostic significance in patients with acute myeloid leukemia. Blood.

[23] Schnittger S, Schoch C, Kern W, Hiddemann W, Haferlach T (2004). FLT3 length mutations as marker for follow-up studies in acute myeloid leukaemia. Acta Haematol.

[24] Kusec R, Jaksic O, Ostojic S, Kardum-Skelin I, Vrhovac R, Jaksic B (2006). More on prognostic significance of FLT3/ITD size in acute myeloid leukemia (AML). Blood.

[25] Ponziani V, Gianfaldoni G, Mannelli F, Leoni F, Ciolli S, Guglielmelli P, Antonioli E, Longo G, Bosi A, Vannucchi AM (2006). The size of duplication does not add to the prognostic significance of FLT3 internal tandem duplication in acute myeloid leukemia patients. Leukemia.

[26] Schneider F, Hoster E, Unterhalt M, Schneider S, Dufour A, Benthaus T, Mellert G, Zellmeier E, Kakadia PM, Bohlander SK, Feuring-Buske M, Buske C, Braess J (2012). The FLT3ITD mRNA level has a high prognostic impact in NPM1 mutated, but not in NPM1 unmutated, AML with a normal karyotype. Blood.

[27] Thol F, Kolking B, Damm F, Reinhardt K, Klusmann JH, Reinhardt D, von Neuhoff N, Brugman MH, Schlegelberger B, Suerbaum S, Krauter J, Ganser A, Heuser M (2012). Next-generation sequencing for minimal residual disease monitoring in acute myeloid leukemia patients with FLT3-ITD or NPM1 mutations. Genes Chromosomes Cancer.

[28] Whitman SP, Archer KJ, Feng L, Baldus C, Becknell B, Carlson BD, Carroll AJ, Mrozek K, Vardiman JW, George SL, Kolitz JE, Larson RA, Bloomfield CD (2001). Absence of the wild-type allele predicts poor prognosis in adult *de novo* acute myeloid leukemia with normal cytogenetics and the internal tandem duplication of FLT3: a cancer and leukemia group B study. Cancer Res.

[29] Kim Y, Lee GD, Park J, Yoon JH, Kim HJ, Min WS, Kim M (2015). Quantitative fragment analysis of FLT3-ITD efficiently identifying poor prognostic group with high mutant allele burden or long ITD length. Blood Cancer J.

[30] Bibault JE, Figeac M, Helevaut N, Rodriguez C, Quief S, Sebda S, Renneville A, Nibourel O, Rousselot P, Gruson B, Dombret H, Castaigne S, Preudhomme C (2015). Next-generation sequencing of FLT3 internal tandem duplications for minimal residual disease monitoring in acute myeloid leukemia. Oncotarget.

[31] Luthra R, Patel KP, Reddy NG, Haghshenas V, Routbort MJ, Harmon MA, Barkoh BA, Kanagal-Shamanna R, Ravandi F, Cortes JE, Kantarjian HM, Medeiros LJ, Singh RR (2014). Next-generation sequencing-based multigene mutational screening for acute myeloid leukemia using MiSeq: applicability for diagnostics and disease monitoring. Haematologica.

[32] Ilyas AM, Ahmad S, Faheem M, Naseer MI, Kumosani TA, Al-Qahtani MH, Gari M, Ahmed F (2015). Next generation sequencing of acute myeloid leukemia: influencing prognosis. BMC Genomics.

[33] Zuffa E, Franchini E, Papayannidis C, Baldazzi C, Simonetti G, Testoni N, Abbenante MC, Paolini S, Sartor C, Parisi S, Marconi G, Cattina F, Bochicchio MT (2015). Revealing very small FLT3 ITD mutated clones by ultra-deep sequencing analysis has important clinical implications in AML patients. Oncotarget.

[34] Frohling S, Scholl C, Levine RL, Loriaux M, Boggon TJ, Bernard OA, Berger R, Dohner H, Dohner K, Ebert BL, Teckie S, Golub TR, Jiang J (2007). Identification of driver and passenger mutations of FLT3 by high-throughput DNA sequence analysis and functional assessment of candidate alleles. Cancer Cell.

[35] Schnittger S, Schoch C, Kern W, Mecucci C, Tschulik C, Martelli MF, Haferlach T, Hiddemann W, Falini B (2005). Nucleophosmin gene mutations are predictors of favorable prognosis in acute myelogenous leukemia with a normal karyotype. Blood.

[36] Braess J, Spiekermann K, Staib P, Gruneisen A, Wormann B, Ludwig WD, Serve H, Reichle A, Peceny R, Oruzio D, Schmid C, Schiel X, Hentrich M (2009). Dose-dense induction with sequential high-dose cytarabine and mitoxantone (S-HAM) and pegfilgrastim results in a high efficacy and a short duration of critical neutropenia in *de novo* acute myeloid leukemia: a pilot study of the AMLCG. Blood.

[37] Schlenk RF, Dohner K, Krauter J, Frohling S, Corbacioglu A, Bullinger L, Habdank M, Spath D, Morgan M, Benner A, Schlegelberger B, Heil G, Ganser A (2008). Mutations and treatment outcome in cytogenetically normal acute myeloid leukemia. N Engl J Med.

[38] Gale RE, Green C, Allen C, Mead AJ, Burnett AK, Hills RK, Linch DC, Medical Research Council Adult Leukaemia Working P (2008). The impact of FLT3 internal tandem duplication mutant level, number, size, and interaction with NPM1 mutations in a large cohort of young adult patients with acute myeloid leukemia. Blood.

[39] Schnittger S, Bacher U, Kern W, Alpermann T, Haferlach C, Haferlach T (2011). Prognostic impact of FLT3-ITD load in NPM1 mutated acute myeloid leukemia. Leukemia.

[40] Quentmeier H, Reinhardt J, Zaborski M, Drexler HG (2003). FLT3 mutations in acute myeloid leukemia cell lines. Leukemia.

[41] McCall CM, Mosier S, Thiess M, Debeljak M, Pallavajjala A, Beierl K, Deak KL, Datto MB, Gocke CD, Lin MT, Eshleman JR (2014). False positives in multiplex PCR-based next-generation sequencing have unique signatures. J Mol Diagn.

[42] Hubmann M, Kohnke T, Hoster E, Schneider S, Dufour A, Zellmeier E, Fiegl M, Braess J, Bohlander SK, Subklewe M, Sauerland MC, Berdel WE, Buchner T (2014). Molecular response assessment by quantitative real-time polymerase chain reaction after induction therapy in NPM1-mutated patients identifies those at high risk of relapse. Haematologica.

[43] Thiede C, Koch S, Creutzig E, Steudel C, Illmer T, Schaich M, Ehninger G (2006). Prevalence and prognostic impact of NPM1 mutations in 1485 adult patients with acute myeloid leukemia (AML). Blood.

[44] Dohner K, Schlenk RF, Habdank M, Scholl C, Rucker FG, Corbacioglu A, Bullinger L, Frohling S, Dohner H (2005). Mutant nucleophosmin (NPM1) predicts favorable prognosis in younger adults with acute myeloid leukemia and normal cytogenetics: interaction with other gene mutations. Blood.

[45] Verhaak RG, Goudswaard CS, van Putten W, Bijl MA, Sanders MA, Hugens W, Uitterlinden AG, Erpelinck CA, Delwel R, Lowenberg B, Valk PJ (2005). Mutations in nucleophosmin (NPM1) in acute myeloid leukemia (AML): association with other gene abnormalities and previously established gene expression signatures and their favorable prognostic significance. Blood.

[46] Leung AY, Man CH, Kwong YL (2013). FLT3 inhibition: a moving and evolving target in acute myeloid leukaemia. Leukemia.

[47] Au CH, Wa A, Ho DN, Chan TL, Ma ES (2016). Clinical evaluation of panel testing by next-generation sequencing (NGS) for gene mutations in myeloid neoplasms. Diagn Pathol.

[48] Bolli N, Manes N, McKerrell T, Chi J, Park N, Gundem G, Quail MA, Sathiaseelan V, Herman B, Crawley C, Craig JI, Conte N, Grove C (2015). Characterization of gene mutations and copy number changes in acute myeloid leukemia using a rapid target enrichment protocol. Haematologica.

[49] Kottaridis PD, Gale RE, Frew ME, Harrison G, Langabeer SE, Belton AA, Walker H, Wheatley K, Bowen DT, Burnett AK, Goldstone AH, Linch DC (2001). The presence of a FLT3 internal tandem duplication in patients with acute myeloid leukemia (AML) adds important prognostic information to cytogenetic risk group and response to the first cycle of chemotherapy: analysis of 854 patients from the United Kingdom Medical Research Council AML 10 and 12 trials. Blood.

[50] Abu-Duhier FM, Goodeve AC, Wilson GA, Gari MA, Peake IR, Rees DC, Vandenberghe EA, Winship PR, Reilly JT (2000). FLT3 internal tandem duplication mutations in adult acute myeloid leukaemia define a high-risk group. Br J Haematol.

[51] Papaemmanuil E, Gerstung M, Malcovati L, Tauro S, Gundem G, Van Loo P, Yoon CJ, Ellis P, Wedge DC, Pellagatti A, Shlien A, Groves MJ, Forbes SA (2013). Clinical and biological implications of driver mutations in myelodysplastic syndromes. Blood.

[52] Schulz WL, Durant TJS, Rinder J, Tormey CA, Torres R, Smith BR, Hager KM, Howe JG, Siddon AJ (2015). Impact of Molecular Clonality on Survival in Acute Myeloid Leukemia. Blood.

[53] Borthakur G, Kantarjian H, Patel KP, Ravandi F, Qiao W, Faderl S, Kadia T, Luthra R, Pierce S, Cortes JE (2012). Impact of numerical variation in FMS-like tyrosine kinase receptor 3 internal tandem duplications on clinical outcome in normal karyotype acute myelogenous leukemia. Cancer.

[54] Spencer DH, Abel HJ, Lockwood CM, Payton JE, Szankasi P, Kelley TW, Kulkarni S, Pfeifer JD, Duncavage EJ (2013). Detection of FLT3 internal tandem duplication in targeted, short-read-length, next-generation sequencing data. J Mol Diagn.

[55] Meshinchi S, Alonzo TA, Stirewalt DL, Zwaan M, Zimmerman M, Reinhardt D, Kaspers GJ, Heerema NA, Gerbing R, Lange BJ, Radich JP (2006). Clinical implications of FLT3 mutations in pediatric AML. Blood.

[56] Ommen HB (2016). Monitoring minimal residual disease in acute myeloid leukaemia: a review of the current evolving strategies. Ther Adv Hematol.

[57] Cloos J, Goemans BF, Hess CJ, van Oostveen JW, Waisfisz Q, Corthals S, de Lange D, Boeckx N, Hahlen K, Reinhardt D, Creutzig U, Schuurhuis GJ, Zwaan Ch M (2006). Stability and prognostic influence of FLT3 mutations in paired initial and relapsed AML samples. Leukemia.

[58] Kronke J, Bullinger L, Teleanu V, Tschurtz F, Gaidzik VI, Kuhn MW, Rucker FG, Holzmann K, Paschka P, Kapp-Schworer S, Spath D, Kindler T, Schittenhelm M (2013). Clonal evolution in relapsed NPM1-mutated acute myeloid leukemia. Blood.

[59] Del Principe MI, Buccisano F, Maurillo L, Sconocchia G, Cefalo M, Consalvo MI, Sarlo C, Conti C, De Santis G, De Bellis E, Di Veroli A, Palomba P, Attrotto C (2016). Minimal Residual Disease in Acute Myeloid Leukemia of Adults: Determination, Prognostic Impact and Clinical Applications. Mediterr J Hematol Infect Dis.

[60] Perl AE, Altman JK, Cortes J, Smith C, Litzow M, Baer MR, Claxton D, Erba HP, Gill S, Goldberg S, Jurcic JG, Larson RA, Liu C (2017). Selective inhibition of FLT3 by gilteritinib in relapsed or refractory acute myeloid leukaemia: a multicentre, first-in-human, open-label, phase 1-2 study. Lancet Oncol.

[61] Altman JK, Perl AE, Cortes JE, Smith CC, Litzow MR, Hi ll JE, Larson RA, Liu C, Ritchie EK, Strickland SA, Wang ES, Neubauer A, Martinelli G (2017). Deep molecular response to gilteritinib to improve survival in FLT3 mutation-positive relapsed/refractory acute myeloid leukemia. Journal of Clinical Oncology.

[62] Bacher U, Haferlach C, Kern W, Haferlach T, Schnittger S (2008). Prognostic relevance of FLT3-TKD mutations in AML: the combination matters—an analysis of 3082 patients. Blood.

[63] MacLeod RA, Kaufmann M, Drexler HG (2007). Cytogenetic harvesting of commonly used tumor cell lines. Nat Protoc.

[64] van Dongen JJ, Macintyre EA, Gabert JA, Delabesse E, Rossi V, Saglio G, Gottardi E, Rambaldi A, Dotti G, Griesinger F, Parreira A, Gameiro P, Diaz MG (1999). Standardized RT-PCR analysis of fusion gene transcripts from chromosome aberrations in acute leukemia for detection of minimal residual disease. Report of the BIOMED-1 Concerted Action: investigation of minimal residual disease in acute leukemia. Leukemia.

[65] Kiyoi H, Naoe T, Yokota S, Nakao M, Minami S, Kuriyama K, Takeshita A, Saito K, Hasegawa S, Shimodaira S, Tamura J, Shimazaki C, Matsue K (1997). Internal tandem duplication of FLT3 associated with leukocytosis in acute promyelocytic leukemia. Leukemia Study Group of the Ministry of Health and Welfare (Kohseisho). Leukemia.

[66] Shih LY, Huang CF, Wu JH, Lin TL, Dunn P, Wang PN, Kuo MC, Lai CL, Hsu HC (2002). Internal tandem duplication of FLT3 in relapsed acute myeloid leukemia: a comparative analysis of bone marrow samples from 108 adult patients at diagnosis and relapse. Blood.

[67] Li H, Handsaker B, Wysoker A, Fennell T, Ruan J, Homer N, Marth G, Abecasis G, Durbin R, 1000 Genome Project Data Processing Subgroup (2009). The Sequence Alignment/Map format and SAMtools. Bioinformatics.

[68] Giardine B, Riemer C, Hardison RC, Burhans R, Elnitski L, Shah P, Zhang Y, Blankenberg D, Albert I, Taylor J, Miller W, Kent WJ, Nekrutenko A (2005). Galaxy: a platform for interactive large-scale genome analysis. Genome Res.

[69] Blankenberg D, Hillman-Jackson J (2014). Analysis of next-generation sequencing data using Galaxy. Methods Mol Biol.

[70] Ye K, Schulz MH, Long Q, Apweiler R, Ning Z (2009). Pindel: a pattern growth approach to detect break points of large deletions and medium sized insertions from paired-end short reads. Bioinformatics.

[71] Cheson BD, Bennett JM, Kopecky KJ, Buchner T, Willman CL, Estey EH, Schiffer CA, Doehner H, Tallman MS, Lister TA, Lo-Coco F, Willemze R, Biondi A (2003). Revised recommendations of the International Working Group for Diagnosis, Standardization of Response Criteria, Treatment Outcomes, and Reporting Standards for Therapeutic Trials in Acute Myeloid Leukemia. J Clin Oncol.

[72] Opatz S, Polzer H, Herold T, Konstandin NP, Ksienzyk B, Zellmeier E, Vosberg S, Graf A, Krebs S, Blum H, Hopfner KP, Kakadia PM, Schneider S (2013). Exome sequencing identifies recurring FLT3 N676K mutations in core-binding factor leukemia. Blood.

