# Supplementary material for: Clonal heterogeneity of FLT3-ITD detected by high-throughput amplicon sequencing correlates with adverse prognosis in acute myeloid leukemia
Source: Oncotarget. 2018 Jul 10;9(53):30128–45. doi: 10.18632/oncotarget.25729 (PMC6059024; doi:10.18632/oncotarget.25729)
Supplement: Supplementary file 1 [file oncotarget-09-30128-s001.pdf]

# Clonal heterogeneity of *FLT3*-ITD detected by high-throughput amplicon sequencing correlates with adverse prognosis in acute myeloid leukemia

## SUPPLEMENTARY MATERIALS

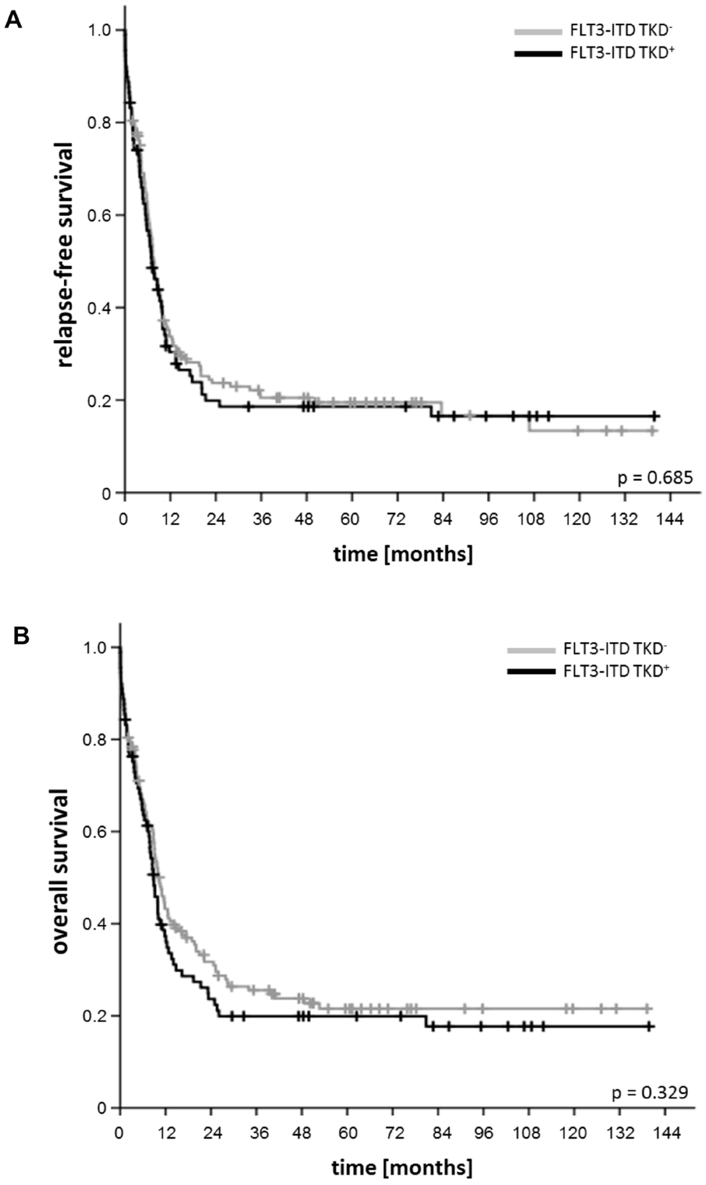


**Supplementary Figure 1: Impact of the occurrence of *FLT3*-ITD in the TKD on RFS and OS.** (**A**) Relapse-free survival (RFS) and (**B**) overall survival (OS) of patients according to harbouring a *FLT3*-ITD inside the TKD (*FLT3*-ITD TKD$^+$) compared to those located in the JM (*FLT3*-ITD TKD$^-$; considering all *FLT3*-ITD clones per patient; $n$ = 242; *FLT3*-ITD TKD$^+$ ($n$ = 89), *FLT3*-ITD TKD$^-$ ($n$ = 153)). ITD (internal tandem duplication), TKD (tyrosine kinase domain).

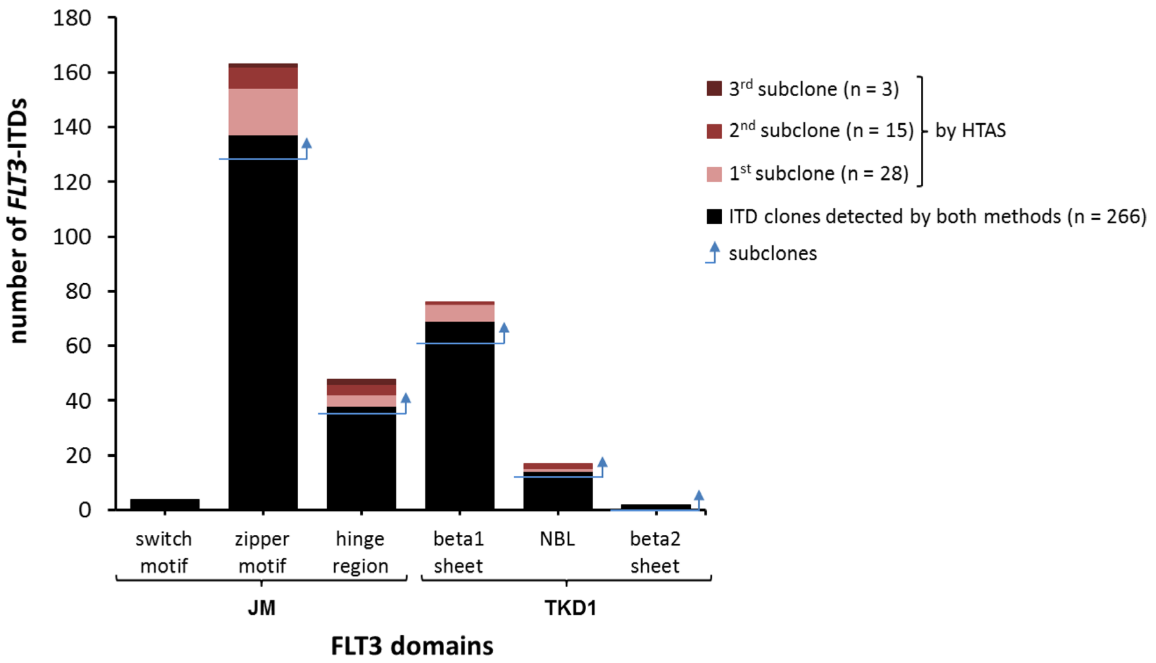


**Supplementary Figure 2: Subclonal ITDs exclusively detected by HTAS according to FLT3 domain.** Of 320 detected ITDs in total (detected by HTAS and/or fragment analysis with cDNA template), 312 ITDs are shown (for the 8 sub-clonal ITDs detected by fragment analysis exclusively no information about ITD position was available). HTAS (high-throughput amplicon sequencing), ITD (internal tandem duplication).

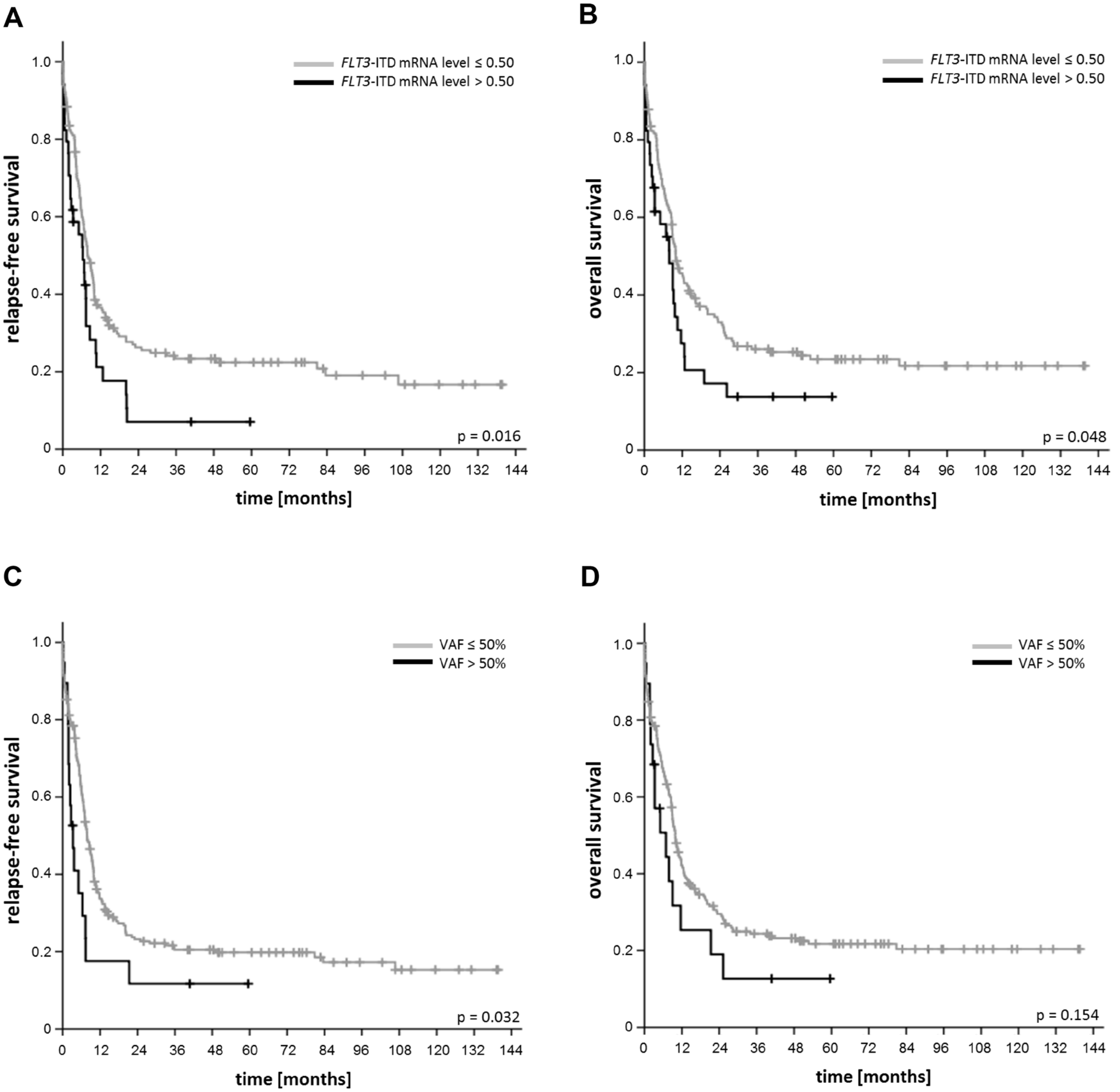


**Supplementary Figure 3: Impact of the *FLT3*-ITD mutational burden of the dominant clone on relapse-free and overall survival.** (**A**) Relapse-free and (**B**) overall survival according to the *FLT3*-ITD mRNA level measured by fragment analysis with cDNA template (*n* =198; *FLT3*-ITD mRNA level ≤ 0.50 (*n* = 164), *FLT3*-ITD mRNA level > 0.50 (*n* = 34)). (**C**) Relapse-free and (**D**) overall survival according to the *FLT3*-ITD variant allele frequency (VAF) measured by HTAS with cDNA template (*n* = 242; VAF ≤ 50% (*n* = 223), VAF > 50% (*n* = 19)). HTAS (high-throughput amplicon sequencing), ITD (internal tandem duplication).

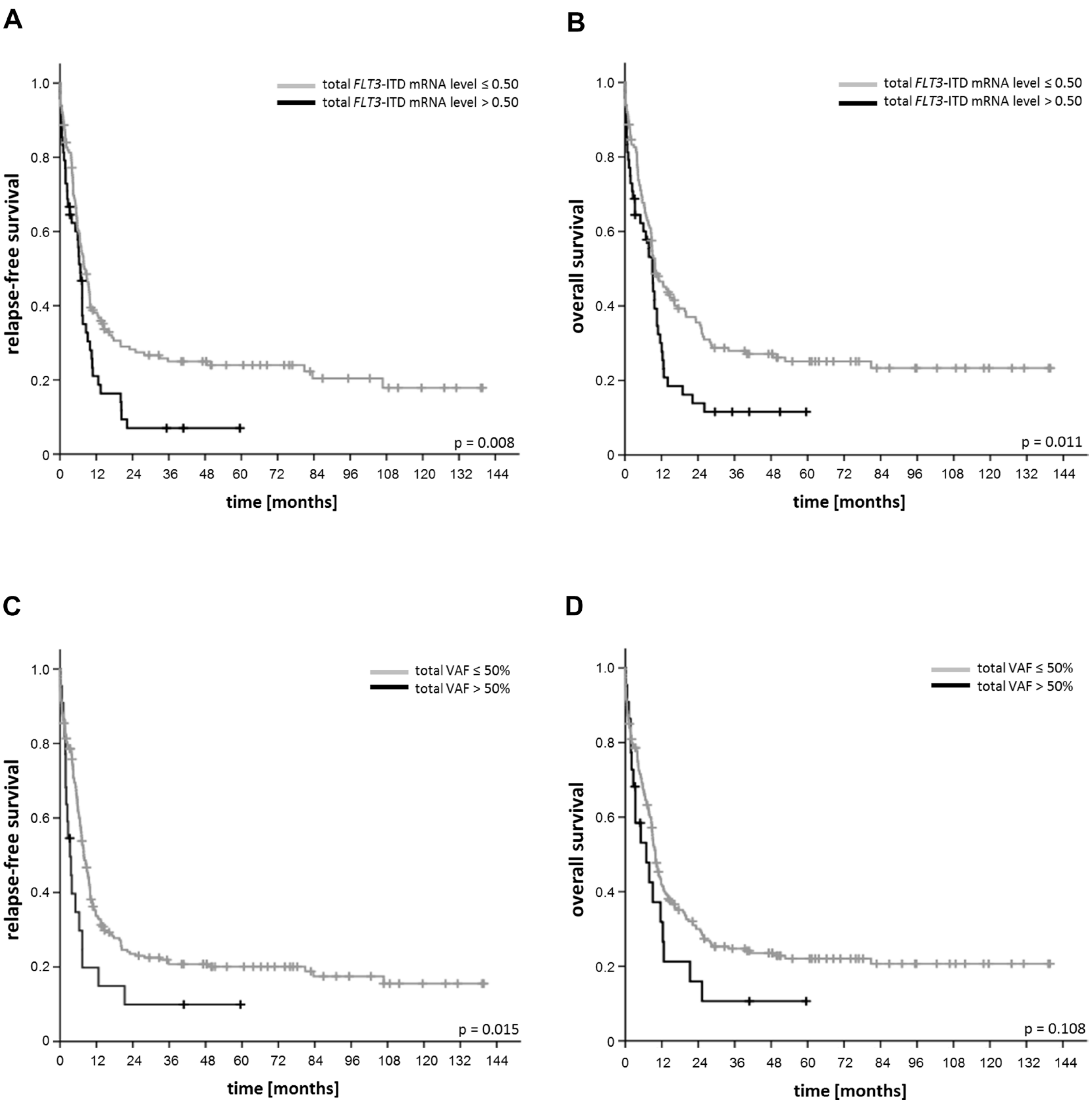


**Supplementary Figure 4: Impact of the *FLT3*-ITD mutational burden of all detected *FLT3*-ITD clones on relapse-free and overall survival.** (**A**) Relapse-free and (**B**) overall survival according to the total *FLT3*-ITD mRNA level of all *FLT3*-ITD clones measured by fragment analysis with cDNA template ($n$ = 198; *FLT3*-ITD mRNA level ≤ 0.50 ($n$ = 150), *FLT3*-ITD mRNA level > 0.50 ($n$ = 48)). (**C**) Relapse-free and (**D**) overall survival according to the total *FLT3*-ITD variant allele frequency (VAF) of all *FLT3*-ITD clones measured by HTAS with cDNA template ($n$ = 242; VAF ≤ 50% ($n$ = 220), VAF > 50% ($n$ = 22)). HTAS (high-throughput amplicon sequencing), ITD (internal tandem duplication).

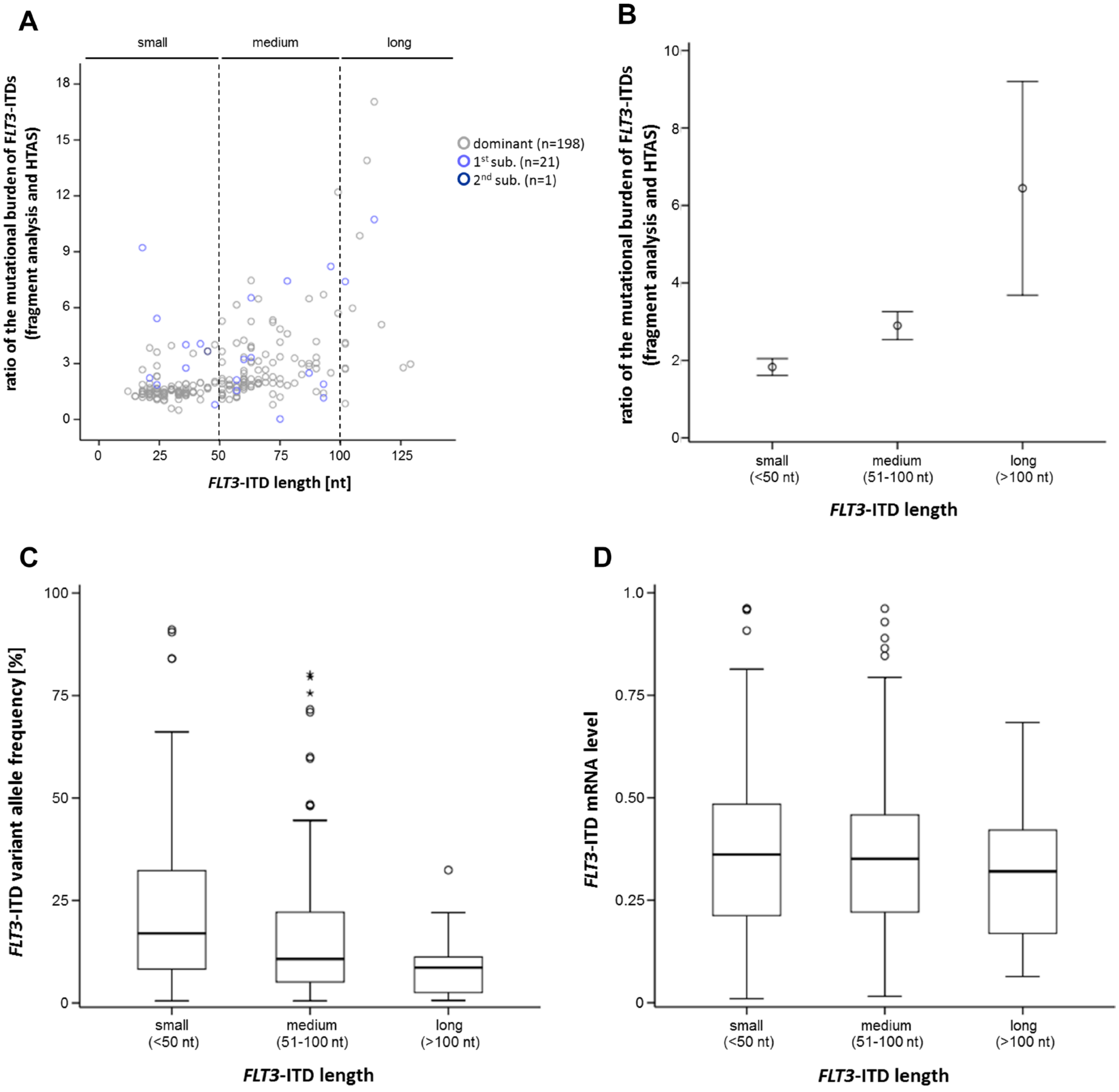


**Supplementary Figure 5: Variance of the mutational burden of *FLT3*-ITD clones according to *FLT3*-ITD size.** Divergence of the mutational burden of *FLT3*-ITDs generated by high-throughput amplicon sequencing (HTAS) and fragment analysis with cDNA template compared to *FLT3*-ITD length (*n* = 220; min. = 12 nt, max. = 129 nt), classified into three groups: small (<50 nt; *n* = 101), median (51–100 nt; *n* = 105) and long (>100 nt; *n* = 14) of (**A**) all data points and (**B**) mean values with the corresponding confidence interval CI(95%). (**C**) Distribution of the mutational burden of *FLT3*-ITDs generated by HTAS clustered according to *FLT3*-ITD size (*n* = 312; small *n* = 141, medium *n* = 151, long *n* = 19). (**D**) Distribution of the mutational burden of *FLT3*-ITDs generated by fragment analysis clustered according to *FLT3*-ITD size (*n* = 228; small *n* = 106, medium *n* = 107, long *n* = 15). nt (nucleotide), ITD (internal tandem duplication), sub (subclone).

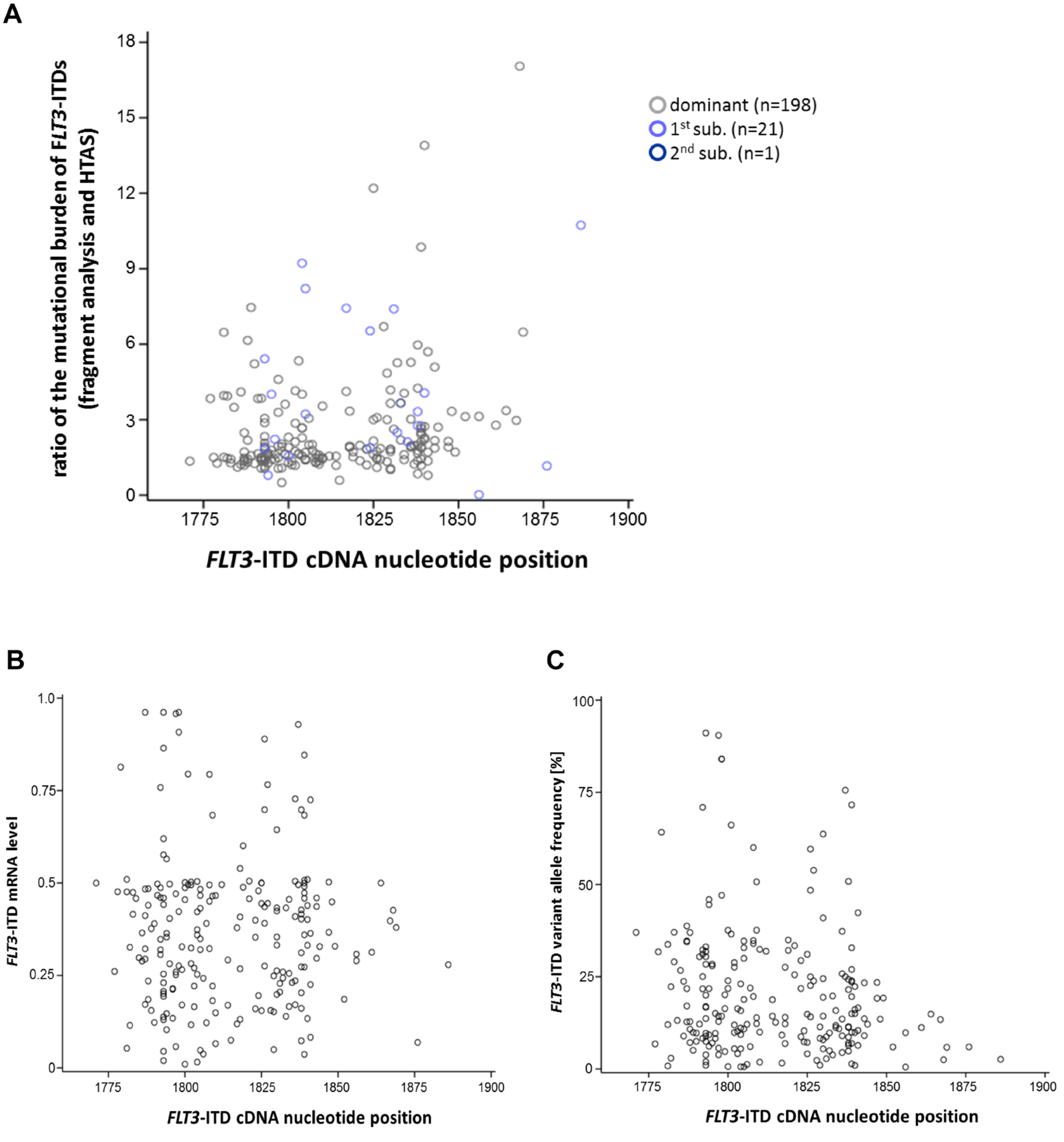


**Supplementary Figure 6: *FLT3*-ITD clone size in relation to *FLT3*-ITD cDNA nucleotide position.** (**A**) Divergence of VAF generated by cDNA-based high-throughput amplicon sequencing (HTAS) and fragment analysis compared to *FLT3*-ITD position (determined by HTAS, $n$ = 220). Correlation of the *FLT3*-ITD cDNA nucleotide position (determined by HTAS) with (**B**) the *FLT3*-ITD mRNA level by fragment analysis and (**C**) the VAF by high-throughput amplicon sequencing (HTAS, $n$ = 220). VAF (variant allele frequency), ITD (internal tandem duplication), sub (subclone).

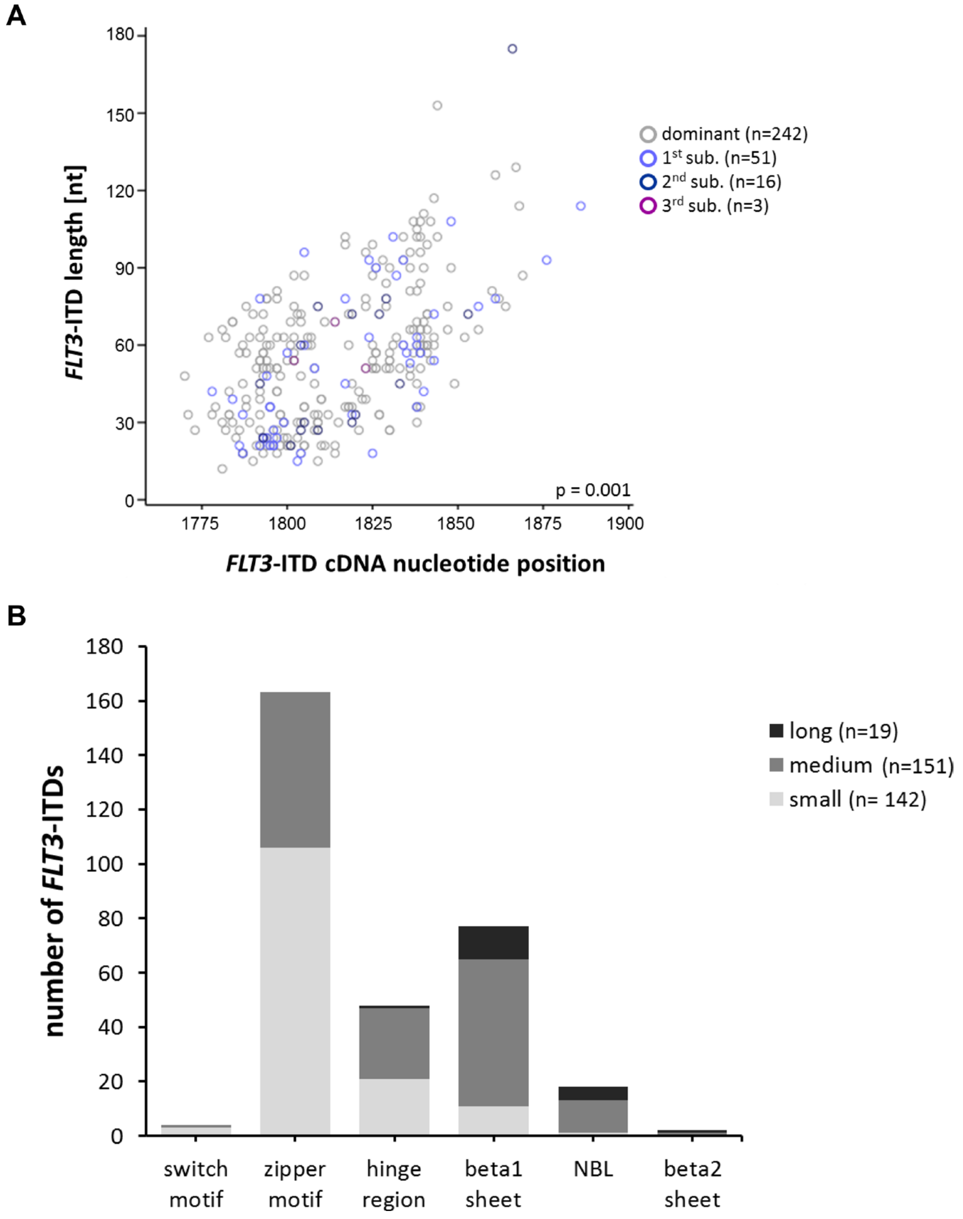


**Supplementary Figure 7: *FLT3*-ITD length in relation to *FLT3*-ITD cDNA nucleotide position determined by HTAS.** (**A**) Localization of ITDs according to cDNA nucleotide position in relation to ITD size, displaying a clustering of ITDs according to clonal size. (**B**) Localization of ITDs according to functional FLT3 domain, grouping ITDs into three groups depending on insertion size (small (<50 nt), medium (51–100 nt), large (>100 nt)). HTAS (high-throughput amplicon sequencing), ITD (internal tandem duplication), sub (subclone).

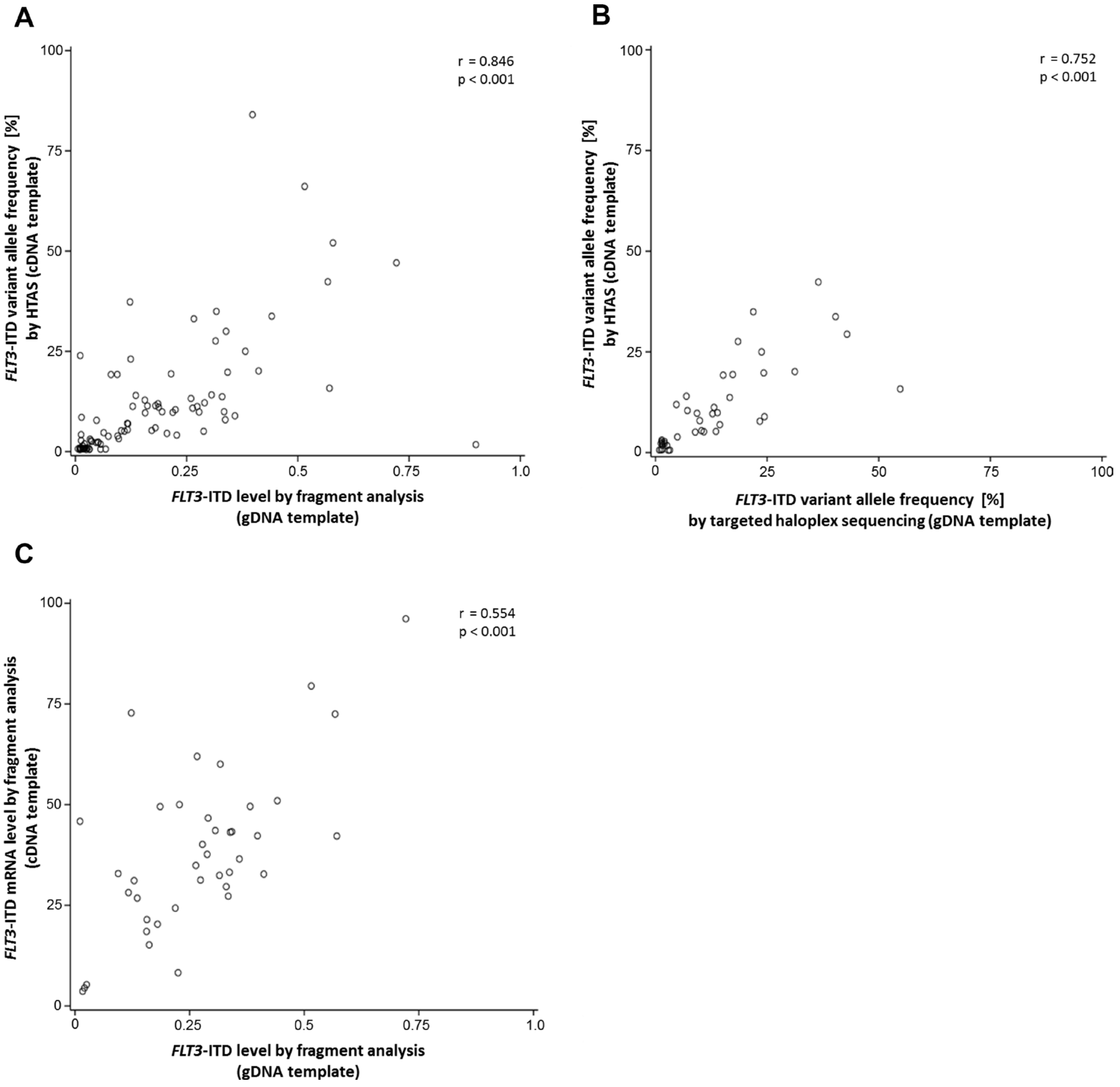


**Supplementary Figure 8: *FLT3*-ITD mutational burden of validated subclones using cDNA versus gDNA template.** Correlation of the variant allele frequency determined by HTAS using cDNA template compared (**A**) to the *FLT3*-ITD level determined by fragment analysis using gDNA template ($n$ = 86 ITDs, in 42 patient samples) and (**B**) to the variant allele frequency determined by targeted haloplex sequencing using gDNA template ($n$ = 41 ITDs, in 22 patient samples). (**C**) Correlation of the *FLT3*-ITD mRNA level by fragment analysis using cDNA template compared to the *FLT3*-ITD level determined by fragment analysis using gDNA template ($n$ = 40 ITDs, in 34 patient samples). HTAS (high-throughput amplicon sequencing), ITD (internal tandem duplication).

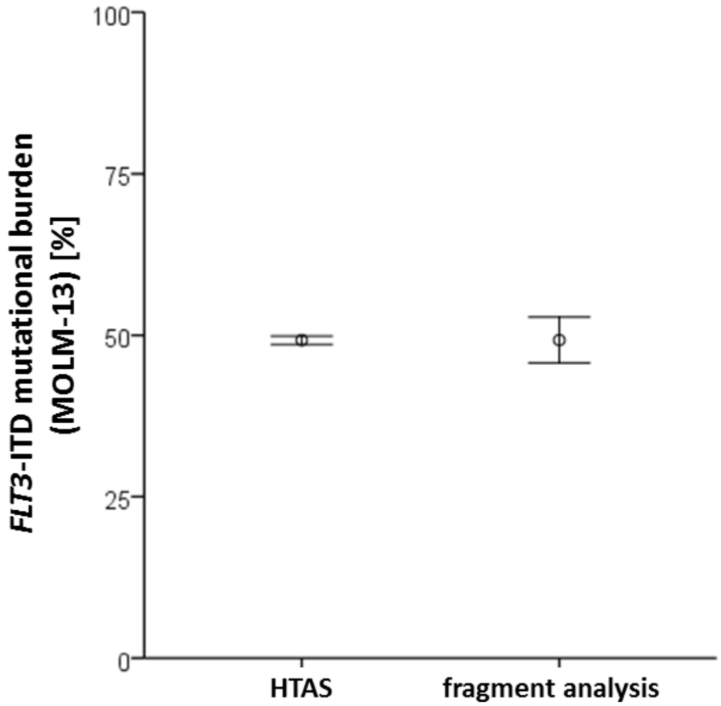


**Supplementary Figure 9: Experimental variance of the *FLT3*-ITD mutational burden of MOLM-13**. Experimental variance of the mutational burden for the 21 nt *FLT3*-ITD detected in MOLM-13 cDNA for both *FLT3*-ITD detection methods high-throughput amplicon sequencing (HTAS) and fragment analysis respectively (*n* = 4; mean values with confidence intervall CI(95%)). ITD (internal tandem duplication), nt (nucleotide).

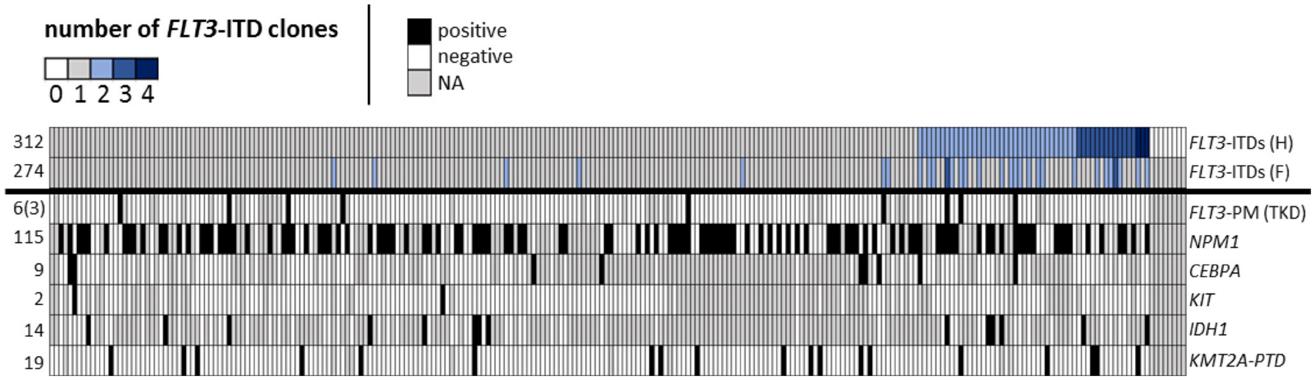


**Supplementary Figure 10: Number of *FLT3*-ITDs and co-occurrence of mutations in other cancer related genes.** PM (point mutations; including D835N and V592L), TKD (tyrosine kinase domain), ITD (internal tandem duplication), H (high-throughput amplicon sequencing; HTAS), F (fragment analysis using cDNA), PTD (partial tandem duplication), NA (not available).

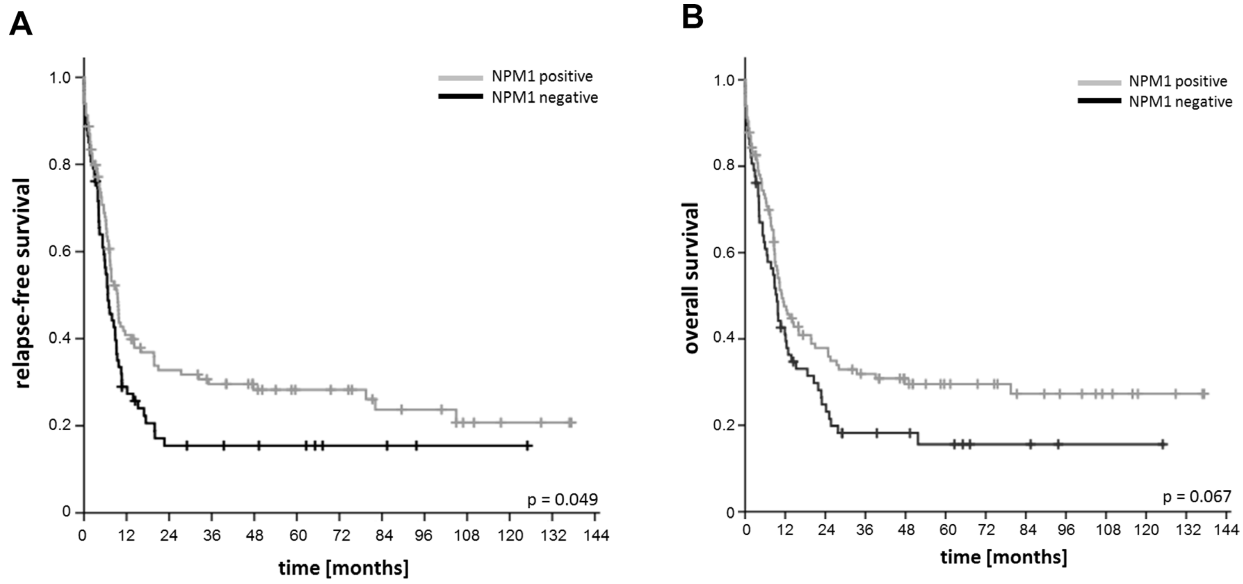


**Supplementary Figure 11: Impact of *NPM1* mutation status on RFS and OS**. (**A**) Relapse-free survival (RFS) and (**B**) overall survival (OS) of *FLT3*-ITD positive patients according to NPM1 mutation ($n$ = 182; NPM1$^{+}$ ($n$ = 115), NPM1$^{-}$ ($n$ = 67)). ITD (internal tandem duplication).

**Supplementary Table 1: *FLT3*-ITDs per patient detected by HTAS and fragment analysis, displaying ITD position, size, length and mutational burden.** See Supplementary_Table_1

**Supplementary Table 2: Validation of *FLT3*-ITD length for cases displaying length differences between HTAS and fragment analysis**

| patient (UPN) | *FLT3*-ITD | | | | | | | | | | | | validated length |
|---|---|---|---|---|---|---|---|---|---|---|---|---|---|
| | **cDNA position** | | | **length [nt]** | | | | | **mutational burden [%]** | | | | |
| | **HTAS** | **S** | **T(g)** | **HTAS** | **F** | **S** | **T(g)** | **F(g)** | **HTAS** | **F** | **T(g)** | **F(g)** | |
| **2** | 1781 | NA | NA | ***66*** | 65 | NA | NA | 66 | 0.82 | 5.30 | NA | 2.53 | HTAS |
| **8** | 1839 | NA | NA | ***66*** | ***63*** | NA | NA | 66 | 1.38 | 3.66 | NA | 1.67 | HTAS |
| **13** | 1861 | 1867 | NA | ***78*** | NA | ***90*** | NA | 78 | 0.92 | NA | NA | 2.82 | HTAS |
| **89** | 1818 | NA | NA | ***60*** | ***66*** | NA | NA | NA | 12.21 | 40.83 | NA | NA | NA |
| **135** | 1832 | 1825 | 1824 | ***87*** | ***78*** | ***78*** | 78 | 78 | 9.77 | 24.30 | 9.30 | 21.88 | F |
| **158** | 1839 | NA | NA | ***51*** | ***45*** | NA | NA | 45 | 23.93 | 45.86 | NA | 1.09 | F |
| **198** | 1793 | NA | NA | ***18*** | ***15*** | NA | NA | 18 | 33.12 | 61.98 | NA | 26.63 | HTAS |
| **200** | 1781 | 1795 | 1794 | ***12*** | ***24*** | ***24*** | 24 | 24 | 33.75 | 50.98 | 40.33 | 44.13 | F |
| **226** | 1795 | 1795 | NA | ***21*** | NA | ***66*** | NA | 66 | 52.06 | NA | NA | 57.90 | F |
| **240** | 1798 | 1843 | NA | ***33*** | ***66*** | ***66*** | NA | 66 | 84.02 | 42.26 | NA | 39.80 | F |
| **249** | (1788) | 1783 | NA | (87) | NA | ***81*** | NA | neg | (0.34) | NA | NA | neg | NA |

Underlined ITD lengths highlight those which were not in-frame. Bold and italic ITD lengths highlight those which were different by HTAS and gDNA fragment analysis/targeted genome sequencing. UPN (unique patient number), HTAS (high-throughput amplicon sequencing), F (fragment analysis using cDNA), S (Sanger sequencing), T(g) (targeted haloplex sequencing using gDNA), F(g) (fragment analysis using gDNA), nt (nucleotide), NA (not available), neg (negative, not detected).

**Supplementary Table 3: Impact of *FLT3*-ITD length on OS, RFS and occurrence with *NPM1*, *KMT2A*-PTD and CEBPA mutations**

| *FLT3*-ITDs | *FLT3*-ITD length [nt] | no. of patients | median OS [days] | hazard ratio | *p*-value | median RFS [days] | hazard ratio | *p*-value | no. of *NPM1*+ (*n* = 115) | no. of *KMT2A*-PTDs+ (*n* = 19) | no. of *CEBPA*+ (*n* = 9) |
|---|---|---|---|---|---|---|---|---|---|---|---|
| **dominant clone by H (*n* = 242)** | <50 | 107 | 288 vs. 264 | 1.021 | 0.891 | 209 vs. 210 | 1.137 | 0.378 | 47 | 3 | 5 |
| | 51–100 | 120 | 258 vs. 289 | 1.062 | 0.685 | 209 vs. 214 | 0.981 | 0.893 | 58 | 16 | 3 |
| | >100 | 15 | 327 vs. 262 | 0.716 | 0.305 | 291 vs. 209 | 0.643 | 0.175 | 10 | 0 | 1 |
| **dominant clone by F (*n* = 242)** | <50 | 106 | 292 vs. 261 | 0.957 | 0.771 | 217 vs. 207 | 1.015 | 0.919 | 48 | 3 | 5 |
| | 51–100 | 121 | 255 vs. 294 | 1.132 | 0.405 | 199 vs. 228 | 1.097 | 0.525 | 57 | 15 | 3 |
| | >100 | 15 | 327 vs. 262 | 0.716 | 0.305 | 291 vs. 209 | 0.643 | 0.175 | 10 | 0 | 1 |

Length of *FLT3*-ITD was assigned according to the dominant clone (*n* = 242) by both methods. OS (overall survival), RFS (relapse-free survival), ITD (internal tandem duplication), PTD (partial tandem duplication), nt (nucleotides), no. (number), H (HTAS, high-throughput amplicon sequencing), F (fragment analysis using cDNA).

**Supplementary Table 4: Impact of FLT3-ITD localization of the dominant clone (*n* = 242) detected by HTAS according to functional domain on CR after induction therapy, RFS and OS**

| survival parameter | FLT3 domain | | | | |
|---|---|---|---|---|---|
| | **JM switch motif (*n* = 4)** | **JM zipper motif (*n* = 128)** | **JM hinge region (*n* = 35)** | **TKD1 beta1-sheet (*n* = 62)** | **NBL (*n* = 13)** |
| **CR after induction therapy (no. (%), $X^2$, *p*-value)** | 2 (50%)<br>0.253<br>0.615* | 70 (61%)<br>0.049<br>0.825* | 20 (67%)<br>0.308<br>0.579* | 38 (63%)<br>0.053<br>0.817* | 6 (55%)<br>0.281<br>0.596* |
| **RFS (HR, *p*-value)** | 1.261<br>0.691** | 0.943<br>0.743** | 1.200<br>0.361** | 1.070<br>0.683** | 0.592<br>0.148** |
| **OS (HR, *p*-value)** | 1.330<br>0.625** | 0.900<br>0.478** | 1.171<br>0.436** | 1.152<br>0.401** | 0.626<br>0.196** |

ITD (internal tandem duplication), TKD1 (tyrosine kinase domain 1), JM (juxtamembrane), NBL (nucleotide binding loop), RFS (relapse-free survival), CR (clinical remission), OS (overall survival), HR (hazard ratio), no. (number). *Chi-Square test **Cox-regression model.

## Supplementary Table 5: Validation of subclonal *FLT3*-ITDs detected by HTAS exclusively

| patient (UPN) | *FLT3*-ITD | | | | | | | | validated |
|---|---|---|---|---|---|---|---|---|---|
| | cDNA position | | length [nt] | | | mutational burden [%] | | | |
| | HTAS | T(g) | HTAS | T(g) | F(g) | HTAS | T(g) | F(g) | |
| **11** | 1795 | neg | ***21*** | neg | ***15*** | 0.50 | neg | 0.99 | (yes) |
| **13** | 1804 | neg | 27 | neg | 27 | 0.81 | neg | 1.09 | yes |
| **13** | 1802 | neg | ***54*** | neg | ***24*** | 0.68 | neg | 0.60 | (yes) |
| **19** | 1839 | NA | 57 | NA | 57 | 2.50 | NA | 3.66 | yes |
| **19** | 1820 | NA | 33 | NA | 33 | 4.52 | NA | 20.57 | yes |
| **24** | 1787 | NA | 33 | NA | neg | 0.94 | NA | neg | no |
| **32** | 1796 | NA | 27 | NA | 27 | 4.70 | NA | 6.37 | yes |
| **49** | 1799 | 1800 | 30 | 30 | 30 | 5.19 | 10.83 | 10.31 | yes |
| **50** | 1838 | 1837 | 60 | 60 | 60 | 5.26 | 13.53 | 17.15 | yes |
| **50** | 1793 | 1794 | 24 | 24 | 24 | 0.79 | 1.56 | 2.15 | yes |
| **50** | 1814 | 1815 | 69 | 69 | 69 | 0.69 | 1.25 | 1.77 | yes |
| **58** | 1786 | 1788 | 21 | 21 | 21 | 6.93 | 14.40 | 11.82 | yes |
| **58** | 1809 | 1810 | 27 | 27 | 27 | 5.09 | 8.90 | 11.03 | yes |
| **71** | 1827 | NA | 72 | NA | 72 | 0.52 | NA | 2.44 | yes |
| **74** | 1819 | 1820 | 33 | 33 | 33 | 2.75 | 2.02 | 3.48 | yes |
| **74** | 1805 | 1806 | 30 | 30 | 30 | 2.31 | 1.52 | 5.12 | yes |
| **77** | 1792 | 1794 | 78 | 78 | 78 | 0.64 | 0.87 | 1.96 | yes |
| **79** | 1819 | NA | 30 | NA | 30 | 0.78 | NA | 1.67 | yes |
| **80** | 1804 | NA | 60 | NA | neg | 0.93 | NA | neg | no |
| **80** | 1823 | NA | 51 | NA | neg | 0.52 | NA | neg | no |
| **83** | 1834 | neg | 60 | neg | neg | 0.54 | neg | neg | no |
| **88** | 1801 | NA | ***21*** | NA | ***12*** | 8.54 | NA | 1.38 | (yes) |
| **95** | 1825 | NA | ***18*** | NA | ***51*** | 0.81 | NA | 0.99 | (yes) |
| **99** | 1803 | NA | 15 | NA | 15 | 3.82 | NA | 7.41 | yes |
| **103** | 1784 | neg | 39 | neg | 39 | 0.89 | neg | 1.09 | yes |
| **105** | 1808 | 1809 | 51 | 51 | 51 | 2.26 | 2.03 | 4.67 | yes |
| **105** | 1819 | 1821 | 72 | 72 | 72 | 1.85 | 1.58 | 5.66 | yes |
| **106** | 1829 | NA | 78 | NA | 78 | 0.64 | NA | 1.38 | yes |
| **116** | 1778 | 1779 | 42 | 42 | 42 | 0.62 | 3.20 | 6.80 | yes |
| **130** | 1817 | neg | 45 | neg | neg | 2.00 | neg | neg | no |
| **130** | 1809 | neg | 75 | neg | neg | 1.53 | neg | neg | no |
| **131** | 1794 | 1797 | 24 | 24 | 24 | 3.11 | 1.40 | 3.29 | yes |
| **137** | 1795 | 1800 | 36 | 36 | 36 | 9.91 | 13.87 | 19.49 | yes |
| **149** | 1792 | NA | 21 | NA | 21 | 4.22 | NA | 1.28 | yes |
| **149** | 1853 | NA | 72 | NA | neg | 0.50 | NA | neg | no |
| **162** | 1771 | 1800 | ***93*** | ***30*** | ***57*** | 2.74 | 1.37 | 1.28 | (yes) |
| **162** | 1792 | 1789 | 45 | 45 | 45 | 0.58 | 2.86 | 3.20 | yes |
| **173** | 1848 | neg | ***108*** | neg | ***105*** | 0.60 | neg | 5.66 | (yes) |
| **182** | 1787 | neg | 18 | neg | NA | 0.96 | neg | NA | no |
| **183** | 1797 | NA | 24 | NA | 24 | 3.23 | NA | 9.75 | yes |
| **206** | 1843 | 1837 | 54 | 54 | 54 | 3.89 | 4.90 | 9.50 | yes |
| **211** | 1836 | neg | 53 | neg | ***165*** | 11.06 | neg | 18.70 | (yes) |
| **211** | 1866 | 1838 | ***175*** | ***175*** | ***174*** | 1.72 | 2.60 | 90.01 | (yes) |
| **218** | 1793 | 1794 | 24 | 24 | 24 | 5.43 | 10.29 | 11.74 | yes |
| **220** | 1826 | NA | ***90*** | NA | ***39*** | 19.21 | NA | 8.00 | (yes) |
| **233** | 1796 | neg | 21 | neg | ***12*** | 0.58 | neg | 3.10 | (yes) |
| **C-1** | 1804 | NA | 30 | NA | neg | 0.58 | NA | neg | no |

Underlined ITD lengths highlight those which were not in-frame. Bold and italic ITD lengths highlight those which were different by HTAS and gDNA fragment analysis/targeted genome sequencing. UPN (unique patient number), C- (control), HTAS (high-throughput amplicon sequencing), T(g) (targeted haloplex sequencing using gDNA), F(g) (fragment analysis using gDNA), nt (nucleotide), NA (not available), neg (negative, not detected).

**Supplementary Table 6: Multivariate analysis of *FLT3*-ITD number and *FLT3*-ITD mutational burden as independent prognostic factor for RFS and OS**

| variable | RFS | | OS | |
|---|---|---|---|---|
| | HR (95% CI) | *p*-value | HR (95% CI) | *p*-value |
| ***FLT3*-ITD number – single clone (H)** | 0.675 (0.450–1.014) | 0.058 | 0.753 (0.500–1.134) | 0.175 |
| ***NPM1* mutation status positive** | 0.626 (0.438–0.896) | 0.010* | 0.666 (0.465–0.954) | 0.027* |
| **normal karyotype** | 0.611 (0.350–1.065) | 0.082 | 0.653 (0.368–1.157) | 0.144 |
| **variable** | **RFS** | | **OS** | |
| | **HR (95% CI)** | ***p*-value** | **HR (95% CI)** | ***p*-value** |
| ***FLT3*-ITD number – single clone (F)** | 0.740 (0.455–1.205) | 0.227 | 0.849 (0.509–1.416) | 0.531 |
| ***NPM1* mutation status positive** | 0.649 (0.455–0.926) | 0.017* | 0.674 (0.470–0.964) | 0.031* |
| **normal karyotype** | 0.593 (0.341–1.031) | 0.064 | 0.634 (0.358–1.120) | 0.117 |
| **variable** | **RFS** | | **OS** | |
| | **HR (95% CI)** | ***p*-value** | **HR (95% CI)** | ***p*-value** |
| **total VAF <50% (H)** | 0.511 (0.284–0.919) | 0.025* | 0.647 (0.361–1.157) | 0.142 |
| ***NPM1* mutation status positive** | 0.609 (0.424–0.872) | 0.007* | 0.648 (0.451–0.931) | 0.019* |
| **normal karyotype** | 0.585 (0.337–1.016) | 0.057 | 0.636 (0.360–1.123) | 0.119 |
| **variable** | **RFS** | | **OS** | |
| | **HR (95% CI)** | ***p*-value** | **HR (95% CI)** | ***p*-value** |
| **total *FLT3*-ITD mRNA level <50% (F)** | 0.565 (0.377–0.845) | 0.005* | 0.615 (0.408–0.928) | 0.020* |
| ***NPM1* mutation status positive** | 0.714 (0.489–1.042) | 0.080 | 0.714 (0.487–1.047) | 0.085 |
| **normal karyotype** | 0.726 (0.403–1.309) | 0.288 | 0.735 (0.400–1.351) | 0.322 |

*n* = 182; *$p$-values < 0.05; H (high-throughput amplicon sequencing), F (fragment analysis using cDNA); RFS (relapse-free survival); OS (overall survival).

**Supplementary Table 7: Exclusion of sequencing non-specific variants detected in the *FLT3* wild-type cell line HL60 and a type of artificial ITD detected in patient samples**

| variant type | cDNA position | sequence | *FLT3*-ITD length [nt] |
|---|---|---|---|
| non-specific variant | 1712 | AAAAGGTAAAAGC | 12 |
| non-specific variant | 1787 | AAAGGTAAAAGCAAAGGTAAAAATTCATTATTCTTTCCTCT ATCTGCAGAACTGCCTATTCCTAACTGACTCATCATTTCATC TCTG | 86 |
| artificial ITD (exemplary) | 1896 | GGTAATCGA**GAATATGAATATGATCTCAAATGGGAGTTT CCAAGAGAAAATTTAGAGTTT**[*] | 59 |
| corresponding dominant clone | 1843 | AGAGAATAT**GAATATGATCTCAAATGGGAGTTTCCAAGA GAAAATTTAGAGTTTGGTAATC**[*] | 60 |

The underlined nucleotide represents the reference base at the given position. *Overlapping sequence between artificial ITD and corresponding dominant clone is displayed in bold.

**Supplementary Table 8: Custom-designed barcode-sequences for next generation *FLT3* amplicon sequencing, enabling a multiplex run of up to 96 samples by primer-combinations**

| | BARCODE | |
|---|---|---|
| **primer no.** | **forward** | **reverse** |
| 1 | ACCA | ACGCCC |
| 2 | AGAT | ATCTGT |
| 3 | CTTC | CGACCT |
| 4 | GGTT | CGGAGC |
| 5 | TATC | CGTTGT |
| 6 | TGCT | CTGTTA |
| 7 | ATAA | GAAAGT |
| 8 | GACC | GCTAAT |
| 9 | NA | GCTGCA |
| 10 | NA | GTCACC |
| 11 | NA | TGACAA |
| 12 | NA | TCGTCA |

NA (not available), no. (number).
