# Supplementary material for: Clonal heterogeneity of FLT3-ITD detected by high-throughput amplicon sequencing correlates with adverse prognosis in acute myeloid leukemia
Source: Oncotarget. 2018 Jul 10;9(53):30128–45. doi: 10.18632/oncotarget.25729 (PMC6059024; doi:10.18632/oncotarget.25729)
Supplement: Supplementary file 2 [file oncotarget-09-30128-s002.docx]

**Supplementary Table 1: *FLT3*-ITDs per patient detected by HTAS and fragment analysis, displaying ITD position, size, length and mutational burden.**

| **patient (UPN)** | ***FLT3*-ITD** | | | | | | | |
| --- | --- | --- | --- | --- | --- | --- | --- | --- |
|  | **cDNA position** | | **length [nt]** | | | **mutational burden [%]** | | **exclusively** |
|  | **HTAS** | **S** | **HTAS** | **F** | **S** | **HTAS** | **F** |  |
| **1** | 1804 | NA | 69 | 69 | NA | 0.59 | 1.57 | no |
| **2** | 1781 | NA | ***66*** | ***65*** | NA | 0.82 | 5.30 | no |
|  | neg | NA | neg | 112 | NA | neg | 6.37 | yes (F) |
| **3** | 1840 | 1843 | 111 | 111 | 111 | 0.96 | 13.34 | no |
| **4** | 1793 | NA | 66 | 66 | NA | 1.01 | 1.96 | no |
|  | 1800 | NA | 57 | 57 | NA | 0.64 | 0.99 | no |
| **5** | 1829 | 1831 | 75 | 75 | 75 | 1.02 | 4.94 | no |
| **6** | 1805 | 1804 | 87 | NA | 87 | 1.12 | NA | no |
| **7** | 1806 | NA | 63 | 63 | NA | 1.23 | 3.75 | no |
| **8** | 1839 | NA | ***66*** | ***63*** | NA | 1.38 | 3.66 | no |
| **9** | 1844 | 1882 | 153 | NA | 153 | 1.62 | NA | no |
| **10** | 1810 | 1813 | 39 | 39 | 39 | 1.85 | 6.54 | no |
| **11** | 1793 | NA | 24 | 24 | NA | 1.92 | 4.47 | no |
|  | 1795 | NA | 21 | neg | NA | 0.50 | neg | yes (H) |
| **12** | 1811 | 1813 | 69 | NA | 69 | 2.01 | NA | no |
| **13** | 1842 | 1843 | 54 | NA | 54 | 2.29 | NA | no |
|  | 1861 | 1867 | ***78*** | NA | ***90*** | 0.92 | NA | no |
|  | 1804 | neg | 27 | NA | neg | 0.81 | NA | yes (H) |
|  | 1802 | neg | 54 | NA | neg | 0.68 | NA | yes (H) |
| **14** | 1828 | 1831 | 93 | 93 | 93 | 2.32 | 15.54 | no |
| **15** | 1794 | 1813 | 24 | 24 | 24 | 21.77 | 35.36 | no |
|  | 1831 | neg | 102 | 102 | neg | 2.77 | 20.51 | no |
| **16** | 1782 | 1783 | 63 | 63 | 63 | 2.92 | 11.50 | no |
| **17** | 1860 | 1867 | 81 | NA | 81 | 3.35 | NA | no |
| **18** | 1797 | NA | 18 | 18 | NA | 3.85 | 5.75 | no |
| **19** | 1825 | 1828 | 99 | 99 | 99 | 4.10 | 50.00 | no |
|  | 1839 | neg | 57 | neg | neg | 2.50 | neg | yes (H) |
|  | 1820 | neg | 33 | neg | neg | 4.52 | neg | yes (H) |
| **20** | 1803 | 1810 | 72 | 72 | 72 | 4.14 | 22.12 | no |
| **21** | 1836 | NA | 51 | 51 | NA | 4.46 | 23.55 | no |
| **22** | 1799 | 1810 | 24 | 24 | 24 | 4.71 | 17.01 | no |
|  | 1876 | neg | 93 | 93 | neg | 5.96 | 6.89 | no |
| **23** | 1832 | 1834 | 63 | 63 | 63 | 4.93 | 25.93 | no |
| **24** | 1789 | NA | 63 | 63 | NA | 5.05 | 37.66 | no |
|  | 1787 | NA | 33 | neg | NA | 0.94 | neg | yes (H) |
| **25** | 1862 | 1864 | 78 | NA | 78 | 5.20 | NA | no |
| **26** | 1794 | 1801 | 78 | NA | 78 | 5.21 | NA | no |
| **27** | 1830 | NA | 51 | 51 | NA | 5.44 | 19.87 | no |

continued on next page

**Supplementary Table 1 continued.**

| **patient (UPN)** | ***FLT3*-ITD** | | | | | | | |
| --- | --- | --- | --- | --- | --- | --- | --- | --- |
|  | **cDNA position** | | **length [nt]** | | | **mutational burden [%]** | | **exclusively** |
|  | **HTAS** | **S** | **HTAS** | **F** | **S** | **HTAS** | **F** |  |
| **28** | 1802 | 1804 | 54 | 54 | 54 | 5.49 | 11.66 | no |
| **29** | 1797 | NA | 78 | 78 | NA | 5.64 | 25.93 | no |
| **30** | 1783 | 1786 | 33 | NA | 33 | 5.80 | NA | no |
| **31** | 1869 | 1870 | 87 | 87 | 87 | 5.86 | 37.97 | no |
| **32** | 1836 | 1837 | 96 | NA | 96 | 5.91 | NA | no |
|  | 1796 | neg | 27 | NA | neg | 4.70 | NA | yes (H) |
| **33** | 1797 | 1798 | 51 | NA | 51 | 5.94 | NA | no |
| **34** | 1852 | 1855 | 63 | 63 | 63 | 5.95 | 18.57 | no |
| **35** | 1807 | 1810 | 60 | 60 | 60 | 6.06 | 12.20 | no |
| **36** | 1794 | 1795 | 24 | 24 | 24 | 6.14 | 10.31 | no |
| **37** | 1841 | NA | 99 | 99 | NA | 6.38 | 36.39 | no |
|  | 1794 | NA | 48 | 48 | NA | 45.95 | 36.39 | no |
|  | neg | NA | neg | 30 | NA | neg | 22.12 | yes (F) |
| **38** | 1838 | 1840 | 60 | 60 | 60 | 6.43 | 27.33 | no |
| **39** | 1794 | 1795 | 78 | NA | 78 | 6.77 | NA | no |
| **40** | 1777 | NA | 63 | 63 | NA | 6.79 | 26.09 | no |
| **41** | 1818 | NA | 36 | 36 | NA | 6.89 | 13.12 | no |
| **42** | 1839 | NA | 108 | 108 | NA | 6.93 | 68.35 | no |
| **43** | 1838 | 1840 | 105 | 105 | 105 | 6.97 | 41.59 | no |
| **44** | 1788 | 1789 | 57 | 57 | 57 | 7.15 | 43.98 | no |
| **45** | 1825 | NA | 60 | 60 | NA | 7.20 | 15.47 | no |
| **46** | 1823 | 1825 | 96 | NA | 96 | 7.29 | NA | no |
| **47** | 1790 | 1792 | 72 | 72 | 72 | 7.48 | 39.02 | no |
|  | 1824 | neg | 63 | 63 | neg | 7.33 | 47.89 | no |
| **48** | 1793 | 1795 | 57 | 57 | 57 | 7.50 | 13.79 | no |
| **49** | 1801 | 1804 | 69 | NA | 69 | 7.79 | NA | no |
|  | 1799 | neg | 30 | NA | neg | 5.19 | NA | yes (H) |
| **50** | 1830 | 1831 | 90 | 90 | 90 | 7.94 | 33.20 | no |
|  | 1838 | neg | 60 | neg | neg | 5.26 | neg | yes (H) |
|  | 1793 | neg | 24 | neg | neg | 0.79 | neg | yes (H) |
|  | 1814 | neg | 69 | neg | neg | 0.69 | neg | yes (H) |
| **51** | 1796 | NA | 27 | 27 | NA | 8.27 | 13.42 | no |
|  | 1805 | NA | 96 | 96 | NA | 0.58 | 4.76 | no |
| **52** | 1829 | 1831 | 84 | 84 | 84 | 8.29 | 24.87 | no |
| **53** | 1792 | 1795 | 63 | 63 | 63 | 8.29 | 31.93 | no |
| **54** | 1838 | 1840 | 102 | 102 | 102 | 8.63 | 7.32 | no |
| **55** | 1831 | 1834 | 60 | 60 | 60 | 8.72 | 22.84 | no |
|  | 1838 | 1840 | 63 | 63 | 63 | 4.85 | 16.11 | no |
| **56** | 1823 | 1825 | 39 | 39 | 39 | 8.73 | 15.97 | no |

continued on next page

**Supplementary Table 1 continued.**

| **patient (UPN)** | ***FLT3*-ITD** | | | | | | | |
| --- | --- | --- | --- | --- | --- | --- | --- | --- |
|  | **cDNA position** | | **length [nt]** | | | **mutational burden [%]** | | **exclusively** |
|  | **HTAS** | **S** | **HTAS** | **F** | **S** | **HTAS** | **F** |  |
| **57** | 1793 | 1795 | 24 | 24 | 24 | 8.89 | 13.04 | no |
| **58** | 1786 | 1789 | 57 | 57 | 57 | 8.91 | 36.51 | no |
|  | 1786 | neg | 21 | neg | neg | 6.93 | neg | yes (H) |
|  | 1809 | neg | 27 | neg | neg | 5.09 | neg | yes (H) |
| **59** | 1836 | 1837 | 66 | 66 | 66 | 8.99 | 17.08 | no |
| **60** | 1843 | 1834 | 117 | 117 | 117 | 9.03 | 45.95 | no |
| **61** | 1817 | NA | 102 | 102 | NA | 9.19 | 37.89 | no |
| **62** | 1794 | NA | 21 | 21 | NA | 9.26 | 14.68 | no |
| **63** | 1804 | 1807 | 48 | 48 | 48 | 9.33 | 37.38 | no |
| **64** | 1817 | 1819 | 99 | NA | 99 | 9.40 | NA | no |
| **65** | 1796 | 1798 | 24 | NA | 24 | 9.60 | NA | no |
| **66** | 1793 | NA | 51 | 51 | NA | 9.67 | 20.06 | no |
| **67** | 1790 | 1792 | 15 | 15 | 15 | 9.72 | 12.28 | no |
| **68** | 1856 | 1858 | 66 | 66 | 66 | 9.83 | 30.75 | no |
| **69** | 1802 | 1804 | 87 | 87 | 87 | 9.87 | 28.21 | no |
| **70** | 1789 | 1792 | 30 | 30 | 30 | 9.91 | 15.54 | no |
| **71** | 1839 | NA | 102 | 102 | NA | 9.93 | 27.27 | no |
|  | 1805 | NA | 60 | 60 | NA | 10.83 | 34.90 | no |
|  | 1827 | NA | 72 | neg | NA | 0.52 | neg | yes (H) |
| **72** | 1810 | NA | 21 | 21 | NA | 9.99 | 14.89 | no |
| **73** | 1803 | NA | 24 | 24 | NA | 10.17 | 16.53 | no |
| **74** | 1841 | 1843 | 72 | 72 | 72 | 10.44 | 8.26 | no |
|  | 1819 | neg | 33 | neg | neg | 2.75 | neg | yes (H) |
|  | 1805 | neg | 30 | neg | neg | 2.31 | neg | yes (H) |
| **75** | 1788 | 1789 | 75 | 75 | 75 | 10.77 | 23.49 | no |
| **76** | 1804 | NA | 72 | 72 | NA | 11.09 | 25.30 | no |
| **77** | 1861 | 1855 | 126 | 126 | 126 | 11.23 | 31.27 | no |
|  | 1792 | neg | 78 | neg | neg | 0.64 | neg | yes (H) |
| **78** | 1834 | 1837 | 102 | 102 | 102 | 11.24 | 45.56 | no |
| **79** | 1829 | 1831 | 51 | 51 | 51 | 11.41 | 15.18 | no |
|  | 1795 | 1798 | 36 | 36 | 36 | 7.02 | 28.16 | no |
|  | 1819 | neg | 30 | neg | neg | 0.78 | neg | yes (H) |
| **80** | 1838 | 1840 | 30 | 30 | 30 | 11.44 | 20.32 | no |
|  | 1796 | 1798 | 21 | 21 | 21 | 9.65 | 21.45 | no |
|  | 1804 | neg | 60 | neg | neg | 0.93 | neg | yes (H) |
|  | 1823 | neg | 51 | neg | neg | 0.52 | neg | yes (H) |
| **81** | 1825 | 1828 | 51 | NA | 51 | 11.66 | NA | no |
| **82** | 1834 | 1837 | 93 | 93 | 93 | 11.81 | 16.60 | no |

continued on next page

**Supplementary Table 1 continued.**

| **patient (UPN)** | ***FLT3*-ITD** | | | | | | | |
| --- | --- | --- | --- | --- | --- | --- | --- | --- |
|  | **cDNA position** | | **length [nt]** | | | **mutational burden [%]** | | **exclusively** |
|  | **HTAS** | **S** | **HTAS** | **F** | **S** | **HTAS** | **F** |  |
| **83** | 1802 | 1804 | 75 | 75 | 75 | 11.93 | 49.52 | no |
|  | 1834 | neg | 60 | neg | neg | 0.54 | neg | yes (H) |
| **84** | 1781 | NA | 30 | 30 | NA | 12.01 | 47.62 | no |
|  | neg | NA | neg | 15 | NA | neg | 7.75 | yes (F) |
| **85** | 1797 | 1798 | 63 | NA | 63 | 12.03 | NA | no |
| **86** | 1806 | NA | 60 | 60 | NA | 12.10 | 24.24 | no |
| **87** | 1844 | 1846 | 102 | 102 | 102 | 12.13 | 32.80 | no |
| **88** | 1791 | 1804 | 21 | 21 | 21 | 12.16 | 46.67 | no |
|  | 1838 | neg | 36 | 36 | neg | 11.27 | 31.13 | no |
|  | 1801 | neg | 21 | neg | neg | 8.54 | neg | yes (H) |
| **89** | 1818 | NA | ***60*** | ***66*** | NA | 12.21 | 40.83 | no |
| **90** | 1809 | 1810 | 27 | 27 | 27 | 12.27 | 18.17 | no |
| **91** | 1799 | 1801 | 63 | NA | 63 | 12.38 | NA | no |
| **92** | 1815 | 1816 | 30 | 30 | 30 | 12.64 | 7.49 | no |
|  | 1793 | neg | 24 | 24 | neg | 3.82 | 20.70 | no |
| **93** | 1787 | 1789 | 18 | 18 | 18 | 12.76 | 17.15 | no |
| **94** | 1784 | 1786 | 69 | NA | 69 | 12.82 | NA | no |
| **95** | 1788 | 1789 | 39 | 39 | 39 | 12.84 | 18.50 | no |
|  | 1825 | neg | 18 | neg | neg | 0.81 | neg | yes (H) |
| **96** | 1797 | NA | 42 | 42 | NA | 12.87 | 25.21 | no |
|  | neg | NA | neg | 66 | NA | neg | 9.17 | yes (F) |
| **97** | 1817 | 1819 | 36 | NA | 36 | 12.93 | NA | no |
| **98** | 1784 | 1786 | 69 | 69 | 69 | 13.15 | 45.83 | no |
| **99** | 1838 | 1837 | 45 | NA | 45 | 13.24 | NA | no |
|  | 1803 | neg | 15 | NA | neg | 3.82 | NA | yes (H) |
| **100** | 1867 | 1864 | 129 | 129 | 129 | 13.40 | 39.76 | no |
| **101** | 1848 | NA | 90 | 90 | NA | 13.50 | 44.90 | no |
| **102** | 1836 | 1837 | 90 | 90 | 90 | 13.54 | 40.93 | no |
| **103** | 1843 | 1846 | 63 | 63 | 63 | 13.63 | 29.63 | no |
|  | 1784 | neg | 39 | neg | neg | 0.89 | neg | yes (H) |
| **104** | 1809 | neg | 30 | 30 | neg | 13.90 | 22.12 | no |
|  | 1824 | 1828 | 93 | 93 | 93 | 10.33 | 19.55 | no |
|  | 1833 | neg | 45 | 45 | neg | 3.81 | 13.94 | no |
| **105** | 1818 | 1819 | 36 | 36 | 36 | 14.01 | 26.79 | no |
|  | 1808 | neg | 51 | neg | neg | 2.26 | neg | yes (H) |
|  | 1819 | neg | 72 | neg | neg | 1.85 | neg | yes (H) |
| **106** | 1826 | 1828 | 51 | 51 | 51 | 14.13 | 43.57 | no |
|  | 1840 | 1843 | 42 | 42 | 42 | 9.87 | 40.12 | no |
|  | 1829 | neg | 78 | neg | neg | 0.64 | neg | yes (H) |

continued on next page

**Supplementary Table 1 continued.**

| **patient (UPN)** | ***FLT3*-ITD** | | | | | | | |
| --- | --- | --- | --- | --- | --- | --- | --- | --- |
|  | **cDNA position** | | **length [nt]** | | | **mutational burden [%]** | | **exclusively** |
|  | **HTAS** | **S** | **HTAS** | **F** | **S** | **HTAS** | **F** |  |
| **107** | 1798 | NA | 21 | 21 | NA | 14.31 | 27.59 | no |
| **108** | 1797 | 1798 | 81 | 81 | 81 | 14.31 | 47.09 | no |
| **109** | 1814 | 1814 | 21 | 21 | 21 | 14.31 | 16.94 | no |
|  | 1835 | neg | 57 | 57 | neg | 10.89 | 23.08 | no |
| **110** | 1830 | 1831 | 54 | 54 | 54 | 14.32 | 26.25 | no |
| **111** | 1825 | 1828 | 87 | 87 | 87 | 14.82 | 44.44 | no |
| **112** | 1864 | NA | 75 | 75 | NA | 14.86 | 50.00 | no |
| **113** | 1839 | NA | 84 | 84 | NA | 14.96 | 35.98 | no |
| **114** | 1840 | 1843 | 60 | 60 | 60 | 14.99 | 33.16 | no |
| **115** | 1796 | 1798 | 21 | 21 | 21 | 15.04 | 21.26 | no |
| **116** | 1804 | 1807 | 18 | 18 | 18 | 15.80 | 42.20 | no |
|  | 1778 | neg | 42 | neg | neg | 0.62 | neg | yes (H) |
| **117** | 1834 | 1837 | 60 | 60 | 60 | 15.84 | 25.54 | no |
| **118** | 1841 | 1843 | 66 | NA | 66 | 15.92 | NA | no |
| **119** | 1840 | 1843 | 90 | 90 | 90 | 16.09 | 43.91 | no |
| **120** | 1803 | 1804 | 30 | 30 | 30 | 16.34 | 27.01 | no |
|  | 1793 | 1795 | 24 | 24 | 24 | 13.67 | 25.37 | no |
| **121** | 1799 | 1801 | 30 | 30 | 30 | 16.35 | 26.56 | no |
| **122** | 1841 | NA | 72 | 72 | NA | 16.56 | 22.53 | no |
| **123** | 1793 | NA | 24 | 24 | NA | 16.71 | 23.02 | no |
| **124** | 1793 | 1795 | 24 | 24 | 24 | 16.92 | 19.35 | no |
|  | 1886 | 1888 | 114 | 114 | 114 | 2.60 | 27.90 | no |
| **125** | 1787 | 1789 | 45 | 45 | 45 | 17.07 | 29.43 | no |
| **126** | 1770 | 1771 | 48 | NA | 48 | 17.09 | NA | no |
| **127** | 1814 | 1816 | 18 | 18 | 18 | 18.77 | 29.18 | no |
| **128** | 1805 | 1807 | 21 | 21 | 21 | 18.83 | 31.97 | no |
|  | 1856 | 1858 | 75 | 75 | 75 | 0.51 | 28.98 | no |
| **129** | 1847 | NA | 75 | 75 | NA | 19.16 | 36.67 | no |
| **130** | 1849 | 1819 | 45 | 45 | 45 | 19.23 | 32.89 | no |
|  | 1817 | neg | 45 | neg | neg | 2.00 | neg | yes (H) |
|  | 1809 | neg | 75 | neg | neg | 1.53 | neg | yes (H) |
| **131** | 1793 | 1795 | 57 | NA | 57 | 19.38 | NA | no |
|  | 1794 | neg | 24 | NA | neg | 3.11 | NA | yes (H) |
| **132** | 1830 | 1831 | 54 | 54 | 54 | 19.42 | 35.40 | no |
| **133** | 1839 | 1840 | 96 | 96 | 96 | 19.62 | 49.11 | no |
| **134** | 1808 | 1810 | 45 | 45 | 45 | 19.65 | 32.25 | no |
| **135** | 1833 | 1834 | 51 | 51 | 51 | 19.78 | 43.28 | no |
|  | 1832 | 1825 | ***87*** | ***78*** | ***78*** | 9.77 | 24.30 | no |
| **136** | 1793 | 1795 | 24 | 24 | 24 | 19.99 | 57.63 | no |

continued on next page

**Supplementary Table 1 continued.**

| **patient (UPN)** | ***FLT3*-ITD** | | | | | | | |
| --- | --- | --- | --- | --- | --- | --- | --- | --- |
|  | **cDNA position** | | **length [nt]** | | | **mutational burden [%]** | | **exclusively** |
|  | **HTAS** | **S** | **HTAS** | **F** | **S** | **HTAS** | **F** |  |
| **137** | 1799 | 1801 | 30 | 30 | 30 | 20.11 | 32.75 | no |
|  | 1795 | neg | 36 | neg | neg | 9.91 | neg | yes (H) |
| **138** | 1838 | 1840 | 69 | 69 | 69 | 21.52 | 42.73 | no |
| **139** | 1792 | 1795 | 27 | 27 | 27 | 21.73 | 30.65 | no |
|  | neg | neg | neg | 96 | neg | neg | 24.59 | yes (F) |
| **140** | 1868 | NA | 114 | 114 | NA | 2.50 | 42.63 | no |
| **141** | 1787 | 1789 | 18 | 18 | 18 | 21.78 | 32.20 | no |
| **142** | 1800 | NA | 24 | 24 | NA | 21.82 | 50.15 | no |
| **143** | 1823 | 1819 | 78 | 78 | 78 | 21.82 | 39.94 | no |
| **144** | 1807 | 1810 | 63 | 63 | 63 | 21.95 | 39.06 | no |
| **145** | 1842 | 1843 | 108 | NA | 108 | 22.05 | NA | no |
| **146** | 1782 | 1783 | 27 | 27 | 27 | 22.31 | 32.61 | no |
| **147** | 1840 | 1843 | 69 | 69 | 69 | 22.34 | 50.93 | no |
| **148** | 1826 | 1828 | 57 | 57 | 57 | 22.62 | 39.10 | no |
| **149** | 1795 | 1798 | 36 | NA | 36 | 23.07 | NA | no |
|  | 1792 | neg | 21 | NA | neg | 4.22 | NA | yes (H) |
|  | 1853 | neg | 72 | NA | neg | 0.50 | NA | yes (H) |
| **150** | 1804 | 1807 | 30 | 30 | 30 | 23.07 | 35.94 | no |
| **151** | 1843 | 1846 | 60 | 60 | 60 | 23.36 | 43.63 | no |
| **152** | 1847 | 1849 | 66 | 66 | 66 | 23.44 | 50.25 | no |
| **153** | 1839 | NA | 60 | 60 | NA | 23.62 | 50.00 | no |
| **154** | 1794 | 1795 | 60 | NA | 60 | 23.64 | NA | no |
| **155** | 1786 | 1789 | 27 | 27 | 27 | 23.74 | 28.88 | no |
| **156** | 1827 | NA | 33 | 33 | NA | 23.87 | 44.48 | no |
|  | neg | NA | neg | 110 | NA | neg | 6.54 | yes (F) |
| **157** | 1800 | 1802 | 54 | 54 | 54 | 23.92 | 45.50 | no |
| **158** | 1839 | NA | ***51*** | ***45*** | NA | 23.93 | 45.86 | no |
| **159** | 1838 | NA | 36 | 36 | NA | 24.27 | 40.26 | no |
| **160** | 1830 | 1831 | 57 | 57 | 57 | 24.50 | 42.43 | no |
| **161** | 1826 | NA | 51 | 51 | NA | 24.98 | 27.54 | no |
|  | 1817 | NA | 78 | 78 | NA | 1.60 | 11.89 | no |
| **162** | 1837 | 1840 | 66 | 66 | 66 | 25.01 | 49.55 | no |
|  | 1834 | neg | 93 | neg | neg | 2.74 | neg | yes (H) |
|  | 1792 | neg | 45 | neg | neg | 0.58 | neg | yes (H) |
| **163** | 1792 | 1795 | 21 | 21 | 21 | 25.05 | 34.77 | no |
| **164** | 1821 | 1822 | 42 | 42 | 42 | 25.53 | 50.52 | no |
| **165** | 1793 | 1795 | 48 | NA | 48 | 25.71 | NA | no |
| **166** | 1836 | NA | 48 | 48 | NA | 25.80 | 50.47 | no |

continued on next page

**Supplementary Table 1 continued.**

| **patient (UPN)** | ***FLT3*-ITD** | | | | | | | |
| --- | --- | --- | --- | --- | --- | --- | --- | --- |
|  | **cDNA position** | | **length [nt]** | | | **mutational burden [%]** | | **exclusively** |
|  | **HTAS** | **S** | **HTAS** | **F** | **S** | **HTAS** | **F** |  |
| **167** | 1785 | NA | 24 | 24 | NA | 26.71 | 29.78 | no |
|  | 1804 | NA | 18 | 18 | NA | 5.47 | 50.42 | no |
| **168** | 1841 | 1843 | 57 | NA | 57 | 26.93 | NA | no |
| **169** | 1822 | 1825 | 39 | NA | 39 | 26.95 | NA | no |
| **170** | 1839 | 1840 | 63 | 63 | 63 | 26.97 | 50.74 | no |
| **171** | 1792 | 1795 | 54 | NA | 54 | 27.30 | NA | no |
| **172** | 1818 | NA | 72 | 72 | NA | 27.58 | 53.92 | no |
| **173** | 1802 | 1804 | 57 | 57 | 57 | 27.59 | 32.43 | no |
|  | 1848 | neg | 108 | neg | neg | 0.60 | neg | yes (H) |
| **174** | 1795 | 1798 | 60 | 60 | 60 | 27.96 | 49.70 | no |
| **175** | 1797 | 1798 | 27 | NA | 27 | 28.11 | NA | no |
| **176** | 1811 | 1812 | 21 | NA | 21 | 28.26 | NA | no |
| **177** | 1793 | 1795 | 72 | 72 | 72 | 28.39 | 86.48 | no |
| **178** | 1795 | NA | 51 | 51 | NA | 28.44 | 40.41 | no |
| **179** | 1802 | 1804 | 63 | 63 | 63 | 28.83 | 50.27 | no |
| **180** | 1783 | 1786 | 42 | 42 | 42 | 29.03 | 41.55 | no |
| **181** | 1809 | 1810 | 30 | NA | 30 | 29.27 | NA | no |
| **182** | 1823 | 1825 | 75 | 75 | 75 | 29.40 | 35.32 | no |
|  | 1787 | neg | 18 | neg | neg | 0.96 | neg | yes (H) |
| **183** | 1805 | 1807 | 36 | 36 | 36 | 29.98 | 43.15 | no |
|  | 1797 | neg | 24 | neg | neg | 3.23 | neg | yes (H) |
| **184** | 1793 | 1795 | 24 | 24 | 24 | 30.42 | 45.95 | no |
| **185** | 1791 | 1792 | 51 | 51 | 51 | 30.42 | 49.75 | no |
| **186** | 1797 | 1798 | 51 | NA | 51 | 31.03 | NA | no |
| **187** | 1825 | NA | 54 | 54 | NA | 31.08 | 50.00 | no |
| **188** | 1792 | 1795 | 33 | 33 | 33 | 31.11 | 46.06 | no |
| **189** | 1778 | 1780 | 33 | 33 | 33 | 31.76 | 47.64 | no |
| **190** | 1793 | NA | 24 | 24 | NA | 31.84 | 44.17 | no |
| **191** | 1812 | NA | 33 | 33 | NA | 31.91 | 49.57 | no |
| **192** | 1819 | NA | 36 | 36 | NA | 32.15 | 48.85 | no |
| **193** | 1792 | NA | 39 | 39 | NA | 32.28 | 48.72 | no |
| **194** | 1837 | 1840 | 108 | NA | 108 | 32.42 | NA | no |
| **195** | 1810 | 1813 | 39 | 39 | 39 | 32.43 | 46.61 | no |
| **196** | 1839 | 1840 | 36 | 36 | 36 | 32.85 | 47.31 | no |
| **197** | 1805 | 1807 | 36 | 36 | 36 | 32.88 | 46.64 | no |
| **198** | 1793 | NA | ***18*** | ***15*** | NA | 33.12 | 61.98 | no |
|  | neg | NA | neg | 48 | NA | neg | 5.66 | yes (F) |
| **199** | 1821 | 1822 | 27 | 27 | 27 | 33.43 | 45.74 | no |
| **200** | 1781 | 1795 | ***12*** | ***24*** | ***24*** | 33.75 | 50.98 | no |

continued on next page

**Supplementary Table 1 continued.**

| **patient (UPN)** | ***FLT3*-ITD** | | | | | | | |
| --- | --- | --- | --- | --- | --- | --- | --- | --- |
|  | **cDNA position** | | **length [nt]** | | | **mutational burden [%]** | | **exclusively** |
|  | **HTAS** | **S** | **HTAS** | **F** | **S** | **HTAS** | **F** |  |
| **201** | 1808 | 1810 | 33 | 33 | 33 | 34.00 | 49.49 | no |
| **202** | 1787 | 1789 | 18 | 18 | 18 | 34.46 | 41.18 | no |
| **203** | 1805 | 1807 | 21 | 21 | 21 | 34.68 | 48.32 | no |
| **204** | 1787 | NA | 18 | 18 | NA | 34.72 | 48.37 | no |
| **205** | 1808 | 1810 | 27 | 27 | 27 | 34.82 | 45.05 | no |
| **206** | 1819 | 1822 | 45 | 45 | 45 | 34.96 | 60.06 | no |
|  | 1843 | neg | 54 | neg | neg | 3.89 | neg | yes (H) |
| **207** | 1801 | 1804 | 21 | 21 | 21 | 36.58 | 49.55 | no |
| **208** | 1788 | 1789 | 36 | 36 | 36 | 36.99 | 48.45 | no |
| **209** | 1771 | NA | 33 | 33 | NA | 37.02 | 50.00 | no |
| **210** | 1783 | NA | 33 | 33 | NA | 37.06 | 47.45 | no |
| **211** | 1836 | 1837 | 81 | 81 | 81 | 37.31 | 72.77 | no |
|  | 1836 | neg | 53 | neg | neg | 11.06 | neg | yes (H) |
|  | 1866 | neg | 175 | neg | neg | 1.72 | neg | yes (H) |
| **212** | 1800 | 1801 | 21 | 21 | 21 | 37.60 | 48.72 | no |
| **213** | 1809 | NA | 15 | 15 | NA | 37.63 | 46.47 | no |
| **214** | 1792 | 1795 | 42 | NA | 42 | 38.60 | NA | no |
| **215** | 1787 | 1789 | 60 | 60 | 60 | 38.76 | 96.15 | no |
| **216** | 1773 | 1774 | 27 | NA | 27 | 39.75 | NA | no |
| **217** | 1830 | 1831 | 27 | 27 | 27 | 40.94 | 43.28 | no |
| **218** | 1841 | 1843 | 60 | 60 | 60 | 42.35 | 72.50 | no |
|  | 1793 | neg | 24 | neg | neg | 5.43 | neg | yes (H) |
| **219** | 1794 | 1795 | 51 | 51 | 51 | 44.55 | 56.52 | no |
| **220** | 1798 | 1801 | 48 | 48 | 48 | 47.08 | 96.15 | no |
|  | 1826 | neg | 90 | neg | neg | 19.21 | neg | yes (H) |
| **221** | 1838 | 1840 | 81 | NA | 81 | 48.06 | NA | no |
| **222** | 1794 | 1795 | 54 | NA | 54 | 48.17 | NA | no |
| **223** | 1826 | 1828 | 57 | 57 | 57 | 48.46 | 69.83 | no |
| **224** | 1809 | NA | 33 | 33 | NA | 50.73 | 68.35 | no |
| **225** | 1838 | 1840 | 36 | 36 | 36 | 50.85 | 69.81 | no |
| **226** | 1795 | 1795 | ***21*** | NA | ***66*** | 52.06 | NA | no |
| **227** | 1827 | 1828 | 33 | 33 | 33 | 53.86 | 76.61 | no |
| **228** | 1784 | 1786 | 30 | NA | 30 | 58.71 | NA | no |
|  | 1843 | 1846 | 72 | NA | 72 | 7.70 | NA | no |
| **229** | 1826 | 1828 | 90 | 90 | 90 | 59.62 | 88.95 | no |
| **230** | 1808 | 1810 | 51 | 51 | 51 | 60.04 | 79.38 | no |
| **231** | 1830 | 1831 | 27 | 27 | 27 | 63.68 | 64.41 | no |
| **232** | 1779 | 1780 | 36 | 36 | 36 | 64.18 | 81.35 | no |

continued on next page

**Supplementary Table 1 continued.**

| **patient (UPN)** | ***FLT3*-ITD** | | | | | | | |
| --- | --- | --- | --- | --- | --- | --- | --- | --- |
|  | **cDNA position** | | **length [nt]** | | | **mutational burden [%]** | | **exclusively** |
|  | **HTAS** | **S** | **HTAS** | **F** | **S** | **HTAS** | **F** |  |
| **233** | 1801 | 1804 | 21 | 21 | 21 | 66.13 | 79.46 | no |
|  | 1796 | neg | 21 | neg | neg | 0.58 | neg | yes (H) |
| **234** | 1792 | NA | 54 | 54 | NA | 70.94 | 75.83 | no |
| **235** | 1839 | 1840 | 57 | 57 | 57 | 71.60 | 84.62 | no |
| **236** | 1837 | NA | 57 | 57 | NA | 75.57 | 92.88 | no |
|  | neg | NA | neg | 24 | NA | neg | 17.50 | yes (F) |
| **237** | 1841 | 1843 | 60 | NA | 60 | 79.49 | NA | no |
| **238** | 1801 | 1804 | 60 | NA | 60 | 80.19 | NA | no |
| **239** | 1798 | NA | 33 | 33 | NA | 84.02 | 90.79 | no |
| **240** | 1798 | 1843 | ***33*** | ***66*** | ***66*** | 84.02 | 42.26 | no |
| **241** | 1797 | 1798 | 42 | 42 | 42 | 90.46 | 95.79 | no |
| **242** | 1793 | 1795 | 24 | 24 | 24 | 91.08 | 96.15 | no |
| **243** | (1840) | NA | (102) | 102 | NA | (0.46) | 1.96 | yes (F) |
| **244** | neg | 1784 | neg | 3 | 3 | neg | 41.42 | yes (F) |
| **245** | neg | NA | neg | 10 | NA | neg | 48.90 | yes (F) |
| **246** | neg | NA | neg | 84 | NA | neg | 6.54 | yes (F) |
| **247** | neg | 1813 | neg | NA | 57 | neg | NA | yes (F) |
| **248** | (1794) | NA | (78) | 78 | NA | (0.22) | 2.25 | yes (F) |
| **249** | (1788) | 1783 | ***(87)*** | NA | ***81*** | (0.34) | NA | yes (F) |
| **250** | (1833) | 1834 | (78) | NA | 78 | (0.35) | NA | yes (F) |
| **C-1** | 1804 | neg | 30 | neg | neg | 0.58 | neg | yes (H) |

Underlined ITD lengths highlight those which were not in-frame. Bold and italic ITD lengths highlight those which were different by HTAS and fragment analysis / Sanger sequencing. Values in light grey and brackets were detected by HTAS below the VAF cut-off level (< 0.5%). UPN (unique patient number), C- (control, assessed *FLT3*-ITD negative according to routine diagnostics), HTAS (high-throughput amplicon sequencing), F (fragment analysis using cDNA), S (Sanger sequencing), nt (nucleotide), NA (not available), neg (negative, not detected).
